# The grapevine homeobox gene *VvHB58* influences seed and fruit development through multiple hormonal signaling pathways

**DOI:** 10.1186/s12870-019-2144-9

**Published:** 2019-11-27

**Authors:** Yunduan Li, Songlin Zhang, Ruzhuang Dong, Li Wang, Jin Yao, Steve van Nocker, Xiping Wang

**Affiliations:** 1 State Key Laboratory of Crop Stress Biology in Arid Areas, College of Horticulture, Northwest A&F University, Yangling, 712100 Shaanxi China; 2 Key Laboratory of Horticultural Plant Biology and Germplasm Innovation in Northwest China, Ministry of Agriculture, Northwest A&F University, Yangling, Shaanxi China; 3 College of Agriculture and Biotechnology, Zhejiang University, Hangzhou, Zhejiang China; 4 Department of Horticulture, Michigan State University, East Lansing, MI USA

**Keywords:** VvHB58, Grapevine, Seed development, Hormones, DNA methylation

## Abstract

**Background:**

The homeobox transcription factor has a diversity of functions during plant growth and development process. Previous transcriptome analyses of seed development in grape hybrids suggested that specific homeodomain transcription factors are involved in seed development in seedless cultivars. However, the molecular mechanism of homeobox gene regulating seed development in grape is rarely reported.

**Results:**

Here, we report that the grapevine *VvHB58* gene, encoding a homeodomain-leucine zipper (HD-Zip I) transcription factor, participates in regulating fruit size and seed number. The *VvHB58* gene was differentially expressed during seed development between seedless and seeded cultivars. Subcellular localization assays revealed that the VvHB58 protein was located in the nucleus. Transgenic expression of *VvHB58* in tomato led to loss of apical dominance, a reduction in fruit pericarp expansion, reduced fruit size and seed number, and larger endosperm cells. Analysis of the cytosine methylation levels within the *VvHB58* promoter indicated that the differential expression during seed development between seedless and seeded grapes may be caused by different transcriptional regulatory mechanisms rather than promoter DNA methylation. Measurements of five classic endogenous hormones and expression analysis of hormone-related genes between *VvHB58* transgenic and nontransgenic control plants showed that expression of *VvHB58* resulted in significant changes in auxin, gibberellin and ethylene signaling pathways. Additionally, several DNA methylation-related genes were expressed differentially during seed development stages in seedless and seeded grapes, suggesting changes in methylation levels during seed development may be associated with seed abortion.

**Conclusion:**

VvHB58 has a potential function in regulating fruit and seed development by impacting multiple hormonal pathways. These results expand understanding of homeodomain transcription factors and potential regulatory mechanism of seed development in grapevine, and provided insights into molecular breeding for grapes.

## Background

Grapevine (*Vitis vinifera*), is one of the most widely cultivated and economically important horticultural fruit crops, with hundreds of cultivars now in use for production of wine, table grapes, and raisins. Over the centuries, seedlessness has been one of the most valued quality traits for table grape cultivars. In recent years, with the increase in consumer demand for seedless grapes, the interest in developing seedless grape cultivars is increasing [1]. Therefore, identifying the genetic mechanisms underlying seedlessness, and the key regulatory genes, are of great significance for seedless grape breeding and meeting the market demand.

Seedless grape cultivars are found in nature and have been preserved via asexual propagation [2]. In seeded genotypes, seed development begins within the ovule after double fertilization, in which an egg cell fuses with a sperm cell to form a diploid embryo, and two polar nuclei fuse with another sperm cell to generate the triploid endosperm. Following fertilization, the ovule develops into a seed. A typical grape berry at maturity contains one to four seeds. Seedlessness can result from either of two mechanisms, parthenocarpy or stenospermocarpy. In parthenocarpy, the stimulus of pollination is sufficient to trigger fruit set. Because the ovary is able to enlarge and form a berry without fertilization, there are no seeds in the fruits [2]. In stenospermocarpy, pollination and fertilization proceed normally, but the embryo aborts, leaving an incompletely formed seed [3]. Stenospermocarpic genotypes are widely used in seedless grape breeding as a hybrid parent material. The most widely accepted hypothesis proposed the inheritance of grape seedlessness controlled by an amino acid substitution in *VviAGL11* is the major cause of seedlessness in grapevine [4–7]. Additionally, grape berry and seed size have a strong genetic component and are thought to be influenced quantitatively by several loci [8–10]. So far, many differentially expressed genes in seedless and seeded grapes have been identified [1, 11]. These genes are mainly involved in seed coat differentiation, hormone homeostasis, epigenetic regulation, reproductive development, cell cycle and primary and secondary metabolism [1, 11]. Because many of these genes may have pleiotropic effects, it is difficult to estimate their specific molecular contribution to seed and fruit development. For instance, the lack of *VviAGL11* expression activation precludes seed coat differentiation and triggers Salicylic acid production along with the overexpression of NAC, Homeobox and WRKY TFs, and eventually leads to endosperm degeneration and embryo developmental arrest [1].

Additionally, hormone signaling is an important biological pathway affecting seed and fruit development. The contents of IAA, GA_3_, ABA and ZR are different between seedless and seeded grapes during seed development stages [11]. Application of GA_3_ to seeded grape inflorescences at pre-bloom induced flower opening and seed abortion. GA_3_-induced morphological alterations may be related to the hormone signaling, alteration of secondary metabolites, the stability of redox homeostasis and regulation of transcription factors [12]. Levels of endogenous hormones also affect fruit size. In the initial stage of grape berry development, berry size increases markedly as auxin and gibberellin directly promote cell division and enlargement. But during grape berry ripening auxin has a negative role. Auxin levels remain low from veraison throughout ripening, and auxin treatment during pre-veraison inhibits berry ripening [13]. Probably, a network coordinated by these hormone levels are regulating the seed and fruit development stages, however, the master regulators that connect all these pathways are still unknown.

In addition, DNA methylation has also been reported to be involved in seed development [14]. During plant growth and development, DNA methylation levels in certain cells or tissues is tightly controlled [15]. In flowering plants, DNA methylation is involved in regulating many epigenetic phenomena mainly including vegetative growth, gene imprinting, gametogenesis, seed development and fruit ripening [15–17]. For example, the DNA glycosylase DEMETER (DME), which catalyzes the elimination of cytosine methylation, is necessary for endosperm gene imprinting and demethylation of maternal alleles, and DME-mediated demethylation is also required for vegetative and central cells in the male gametophyte [18]. DNA methylation reprogramming is also required for embryogenesis. After fertilization, compared with aerial tissues and endosperm, MET1, CMT3, and RdDM-mediated pathways are highly active, leading to a global hypermethylation in the torpedo to mature green stage embryo [19, 20]. Furthermore, inhibition of DNA cytosine methylation accelerated fruit ripening in tomato, and uncoupled seed maturation from fruit development [21]. However, during embryogenesis and seed development there have been few reports on the regulatory mechanism of DNA methylation in grapevine. Therefore, the potential influence of DNA methylation on seed development in grape remains to be further studied.

Homeobox (HB) genes are found among higher eukaryotes and are characterized by a conserved, ~ 180-nucleotide ‘homeobox’ sequence that encodes a ~ 60-amino acid DNA-binding domain, called the homeodomain (HD). HD-containing proteins have been best characterized as transcription factors. In plants, HD transcription factors play an important role in various aspects of plant growth and development, including embryo patterning and vascular development [22], floral organogenesis, fruit ripening [23], seed development [24], cell differentiation and shoot apical meristem maintenance [25], and response to auxin [26]. Transcriptome analyses of seed development in grape hybrids and expression analyses of the homeobox genes during seed development in seedless and seeded grapes have revealed a part of homeobox genes may be involved in seed development process [11, 27]. Three members of the HD-Zip I subfamily, *VvHB62*, *VvHB54* and *VvHB7*, exhibited remarkable differences in the expression pattern during seed development between seeded and seedless grape cultivars, with relatively low expression levels in seeded cultivars [28]. Additionally, two members of the WOX family (a subfamily of the homeobox gene family), *VvWOX3* and *VvWOX11*, were strongly activated during the torpedo and cotyledonary stages of somatic embryogenesis, but showed a relatively low expression level in the earlier developmental stages [29].

The goal of this study was to characterize a potential role for another grapevine HD-Zip I gene, *VvHB58*, in seed and fruit development process. This gene was targeted based on its phylogenetic relationships with other important HB genes and its differential expression during seed development. Through analyzing *VvHB58* function in transgenic tomato, we obtained preliminary evidence that *VvHB58* gene regulates seed and fruit development. Furthermore, differential expression of DNA methylation-related genes during seed development in seeded and seedless cultivars suggests DNA methylation may play a potential role in seed development in grapevine. Taken together, these results will provide a basis for further studying the regulatory mechanism of seed and fruit development in grapevine.

## Results

### Expression pattern and subcellular localization of the VvHB58 protein

**Fig. 1:**
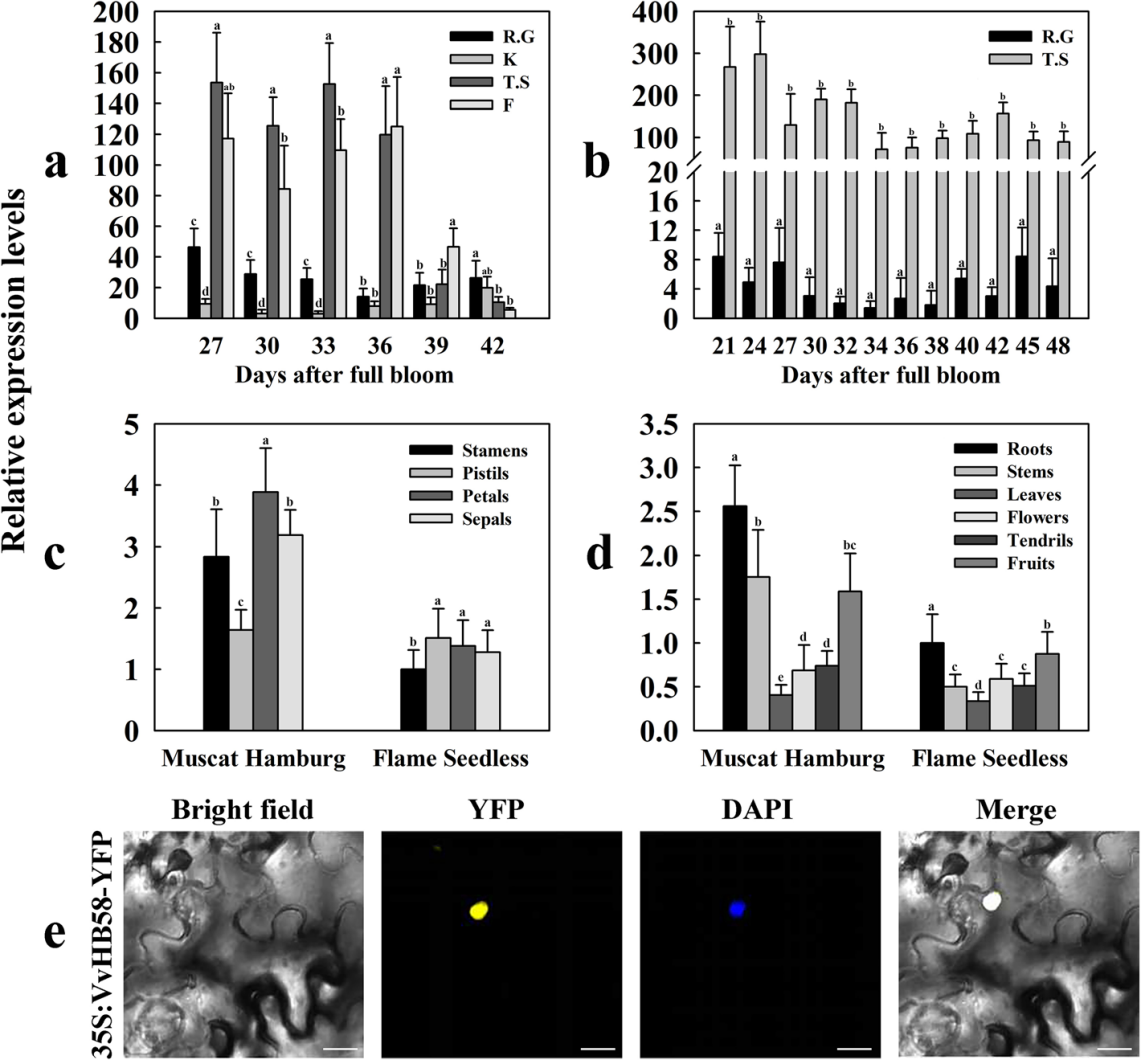
Real-time quantitative RT-PCR analysis of *VvHB58* gene expression levels and subcellular localization of the VvHB58 protein. **a** Expression levels of *VvHB58* during different seed developmental stages from 3-year-old seedless grape cultivars ‘Thompson Seedless’ (T.S) and ‘Flame Seedless’ (F), and seeded grape cultivars ‘Red Globe’ (R.G) and ‘Kyoho’ (K). **b** Expression levels of the *VvHB58* gene during different seed developmental stages from 4-year-old ‘Thompson Seedless’ and ‘Red Globe’. **c** Expression levels of the *VvHB58* gene in different reproductive organs (stamen, pistil, petal and sepal) from 3-year-old seedless cultivar ‘Flame Seedless’ and seeded grape cultivar ‘Muscat Hamburg’. **d** Expression levels of the *VvHB58* gene in different vegetative organs (root, stem, leaf, flower, tendril and fruit) from 3-year-old ‘Flame Seedless’ and ‘Muscat Hamburg’. The grapevine *Actin* (GenBank Accession number: AY680701) and *GAPDH* (GenBank Accession number: CB973647) were used as an endogenous control to normalize expression. Bars represent means ± SD from three biological replicates. Different letters indicate statistically significant differences (Dunn’s test; *P* < 0.05). **e** Subcellular localization of the VvHB58 protein in tobacco leaves. YFP, yellow fluorescent protein. DAPI, 4,6-diamidino-2-phenylindole dihydrochloride. Bars, 100 μm

Previous studies analyzed the seed transcriptomes of progeny derived from the seedless paternal parent ‘Centennial seedless’ (*V. vinifera*) and seeded maternal parent ‘Red Globe’ (*V. vinifera*) to identify genes related to seed development. Transcriptome data revealed some homeobox genes may be involved in seed development process, and *VvHB58* gene (Accession number: GSVIVT01008065001; GenBank: CBI15277) showed a differential expression pattern in the seeds of seedless and seeded progeny [11]. In this study, we used another four grape cultivars (two 3-year-old seedless cultivars ‘Thompson Seedless’ and ‘Flame Seedless’, and two 3-year-old seeded cultivars ‘Red Globe’ and ‘Kyoho’) to verify the expression pattern of *VvHB58*, so as to explore whether this differential expression pattern is universal. Previous studies have reported that the mass of the seeds in seedless grapes generally begins to decrease at 27 - 33 days after full bloom (DAF) [11]. We used qPCR to analyze the expression levels of *VvHB58* during seed development at 27, 30, 33, 36, 39 and 42 DAF in these four cultivars (Fig. 1a). *VvHB58* showed higher expression in the two seedless cultivars relative to the seeded cultivars. In order to further explore the expression pattern of *VvHB58* in seed development stages, we selected 12 seed development stages to evaluate the expression of *VvHB58* in 4-year-old ‘Thompson Seedless’ and ‘Red Globe’ (Because ‘Thompson Seedless’ and ‘Red Globe’ are representative cultivars of seedless grapes and seeded grapes respectively, the two cultivars were further considered for expression analysis in the following year). During seed development between 21 to 48 DAF, expression of *VvHB58* was high in ‘Thompson Seedless’, but relatively low in ‘Red Globe’ (Fig. 1b), suggesting that *VvHB58* may participate in seed development of seedless grapes.

In addition, we analyzed the expression pattern of *VvHB58* in floral organs (stamen, pistil, petal and sepal) (Fig. 1c) and in the whole flower, root, stem, leaf, tendril and fruit (Fig. 1d) in the seedless cultivar ‘Flame Seedless’ and the seeded cultivar ‘Muscat Hamburg’. In general, the expression levels of *VvHB58* in both vegetative and reproductive organs were higher in ‘Muscat Hamburg’ than in ‘Flame Seedless’, especially in the stamens, petals, sepals, roots, stems and fruit.

Consistent with its presumed role as a transcription factor, VvHB58 contains a putative nuclear localization sequence (NLS) of 10 amino acids, located at the beginning of the homeodomain (amino acids 68–78) (http://nls-mapper.iab.keio.ac.jp/cgi-bin/NLS_Mapper_form.cgi). To determine if VvHB58 might be localized in the nucleus, we fused the VvHB58 coding sequence to Yellow Fluorescent Protein (YFP) and expressed this transiently in tobacco (*Nicotiana tabacum*). As shown in Fig. 1e, the VvHB58-YFP protein accumulated in the nucleus.

### Evolutionary analysis of VvHB58 protein

**Fig. 2:**
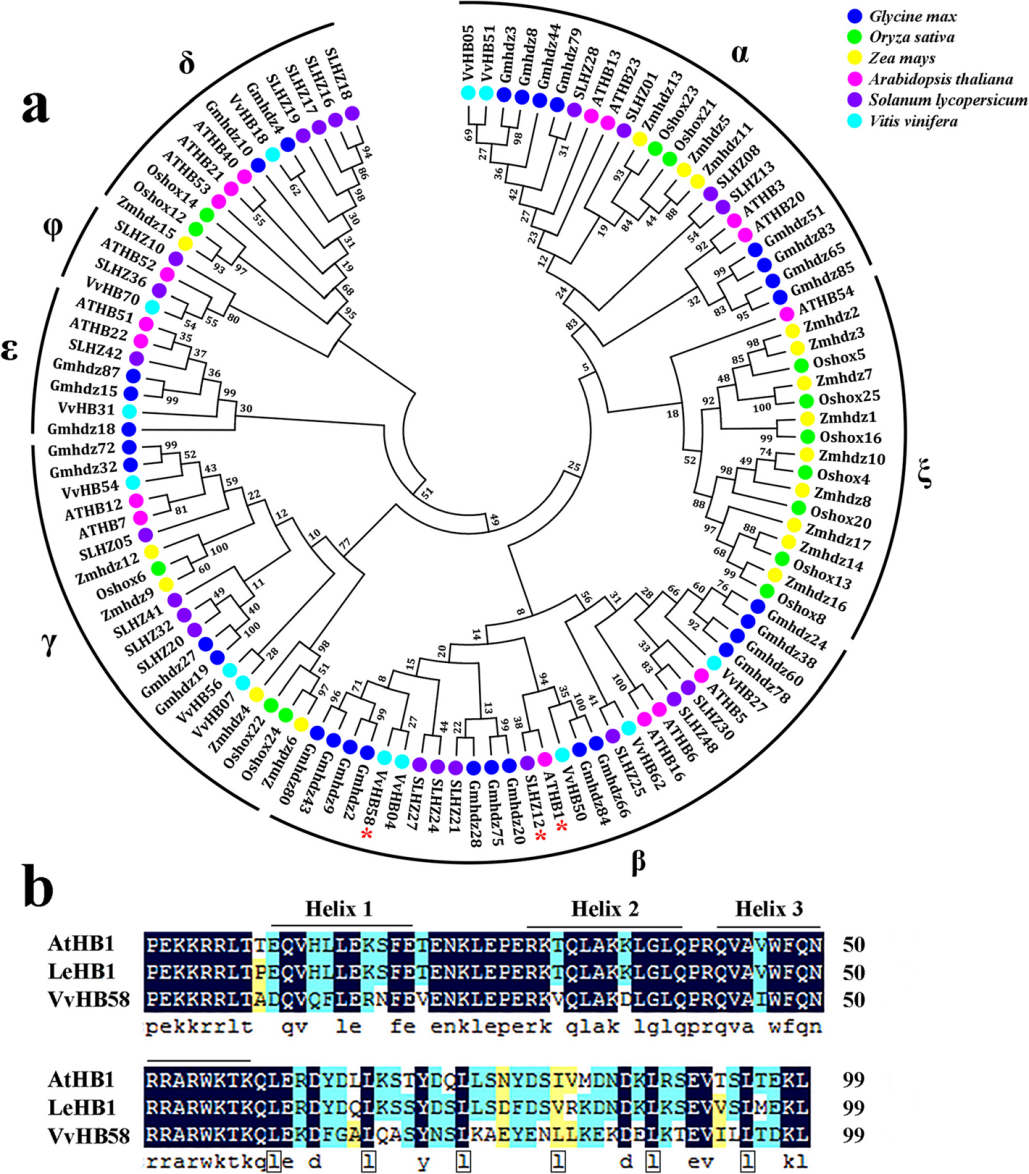
Sequence analysis of the VvHB58 protein. **a** Phylogenetic analysis of HD-Zip I proteins in grapevine (*Vitis vinifera*), tomato (*Solanum lycopersicum*), Arabidopsis (*Arabidopsis thaliana*), rice (*Oryza sativa*), soybean (*Glycine max*) and maize (*Zea mays*). The phylogenetic tree was constructed with MEGA 6.0 by the Neighbor-Joining method and 1000 bootstrap replicates using the full-length protein sequences. Different colored circles represent different species. GenBank accession numbers for proteins are given in Additional file 1: Table S1. **b** Amino acid sequence alignment analysis of the conserved homeodomain region of VvHB58 with its closest homologs from Arabidopsis and tomato. Sequence alignment was performed using DNAMAN software. LeHB1 is also known as SLHZ12

We previously reported the genome-wide identification of the homeodomain transcription factor family in the grapevine genome [27, 28]. An analysis of phylogeny and conserved domains identified members of the HD-Zip I family, including *VvHB58*. In this study, we further explored the evolutionary relationship between VvHB58, other grapevine HD-Zip I proteins, and HD-Zip I proteins from tomato [30], Arabidopsis [31], rice [32], soybean [33] and maize [34] (Additional file 1: Table S1). These HD-Zip I proteins can be divided into seven subfamilies: α, β, γ, δ, ε, ξ and φ (Fig. 2a). Interestingly, most HD-Zip I proteins from monocots including rice and maize clustered together in the ξ subgroup, while HD-Zip I proteins from dicots including Arabidopsis, tomato, soybean and grapevine were generally clustered together. The protein most homologous to VvHB58 in Arabidopsis is ATHB1, which has been reported to be involved in leaf development [35]. A homologous protein in tomato, LeHB1, has been reported to participate in fruit ripening and floral organogenesis [23]. Sequence alignment revealed that the homeodomain of VvHB58 shares 82.5% similarity with those of ATHB1 and LeHB1 (also known as SLHZ12) (Fig. 2b). The complete coding sequence of VvHB58 was amplified from seeds cDNA from the seedless grape ‘Thompson Seedless’ and the seeded grape ‘Red Globe’. This confirmed that genes from two cultivars encode identical proteins of 263 amino acids with a highly conserved homeodomain (amino acids 71 - 124) and a leucine zipper (amino acids 126 - 167).

### Heterologous expression of *VvHB58* in tomato causes a decrease in fruit size

**Fig. 3:**
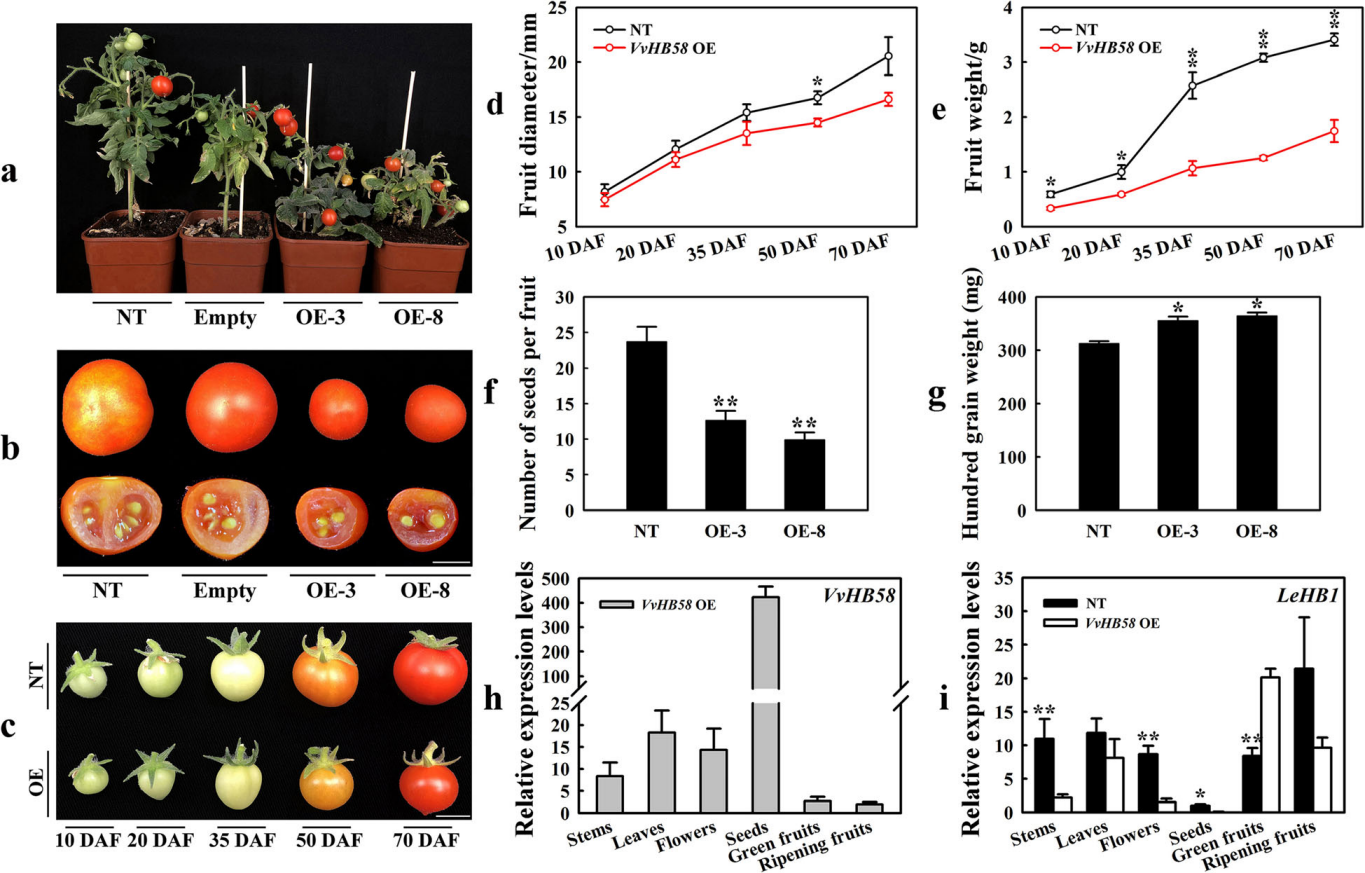
Phenotype of nontransgenic and *VvHB58*-expressing transgenic tomatoes. **a** Phenotype of a representative non-transgenic plant (NT), control transgenic plant transformed with an empty vector, and transgenic lines OE-3 and OE-8. **b** Ripening fruits. Bars, 1 cm. **c** Fruit at 10, 20, 35, 50 and 70 DAF from non-transgenic and transgenic lines. Bars, 1 cm. **d** Fruit diameter (mm). **e** Fruit weight (g). **f** The number of seeds per fruit. The number of seeds in 12 tomatoes per genotype was quantified. **g** Hundred grain weight (mg) of transgenic and non-transgenic tomatoes seeds. The data represents the means +/-SE for 18 fruits, from three plants, and six fruits collected per plant. Significant differences were calculated by one-way ANOVA and are indicated by * (0.01 < *P* value < 0.05) and ** (*P* value < 0.01). **h** Expression analysis of *VvHB58*. **i** Expression analysis of *LeHB1*. Data represent the means +/-SD from three independent biological repeats. Asterisks represent statistical significance (*0.01 < *P* < 0.05, **P < 0.01, one-way ANOVA)

To further investigate a potential role for *VvHB58* in seed development, we expressed the *VvHB58* gene in transgenic tomato (Fig. 3). Compared with nontransgenic tomatoes and a transgenic control only containing the empty vector, *VvHB58* transgenic tomatoes were obviously dwarfed and exhibited reduced apical dominance (Fig. 3a). At the fruit ripening stage, transgenic *VvHB58* lines showed smaller fruits with decreased seed number, but larger seeds (Fig. 3b-g). The number of *VvHB58* transgenic tomatoes seeds was about half of that of non-transgenic tomatoes (Fig. 3f), but the weight of the transgenic tomatoes seeds increased slightly (Fig. 3g). It is speculated that *VvHB58* may play a role in seed development. Although the number and weight of transgenic tomatoes seeds changed, the seeds developed normally. Additionally, we further analysed the fruit diameters and weights of transgenic and nontransgenic fruits during the fruit development and ripening process (Fig. 3c-e). During 10 - 70 DAF (from fruit set development to fruit ripening), both fruit diameters and weights of transgenic fruits were significantly smaller than nontransgenic type, suggesting *VvHB58* gene may affect the early development of fruit sets, leading to a reduction of final cell number in fruits. Between 20 and 35 DAF, the weight of fruit from nontransgenic tomatoes increased rapidly, but that of fruit from *VvHB58* transgenic lines increased only slowly.

Additionally, we analyzed the expression levels of *VvHB58* and *LeHB1* (the tomato homolog of *VvHB58*) in transgenic and non-transgenic tomatoes. *VvHB58* was expressed especially strongly in seeds of the transgenic tomatoes. This is similar to the strong expression of *VvHB58* seen during the seed development of seedless grapes (Fig. 3h). The transcript levels of *LeHB1* gene involved in floral organogenesis and fruit ripening were not generally increased in the transgenic tomatoes (Fig. 3i), and *LeHB1* gene only showed a higher transcript level in green fruits of transgenic tomatoes, suggesting ectopic expression of *VvHB58* gene in transgenic tomatoes didn’t result in increased expression of it’s homologous genes in tomatoes.

**Fig. 4:**
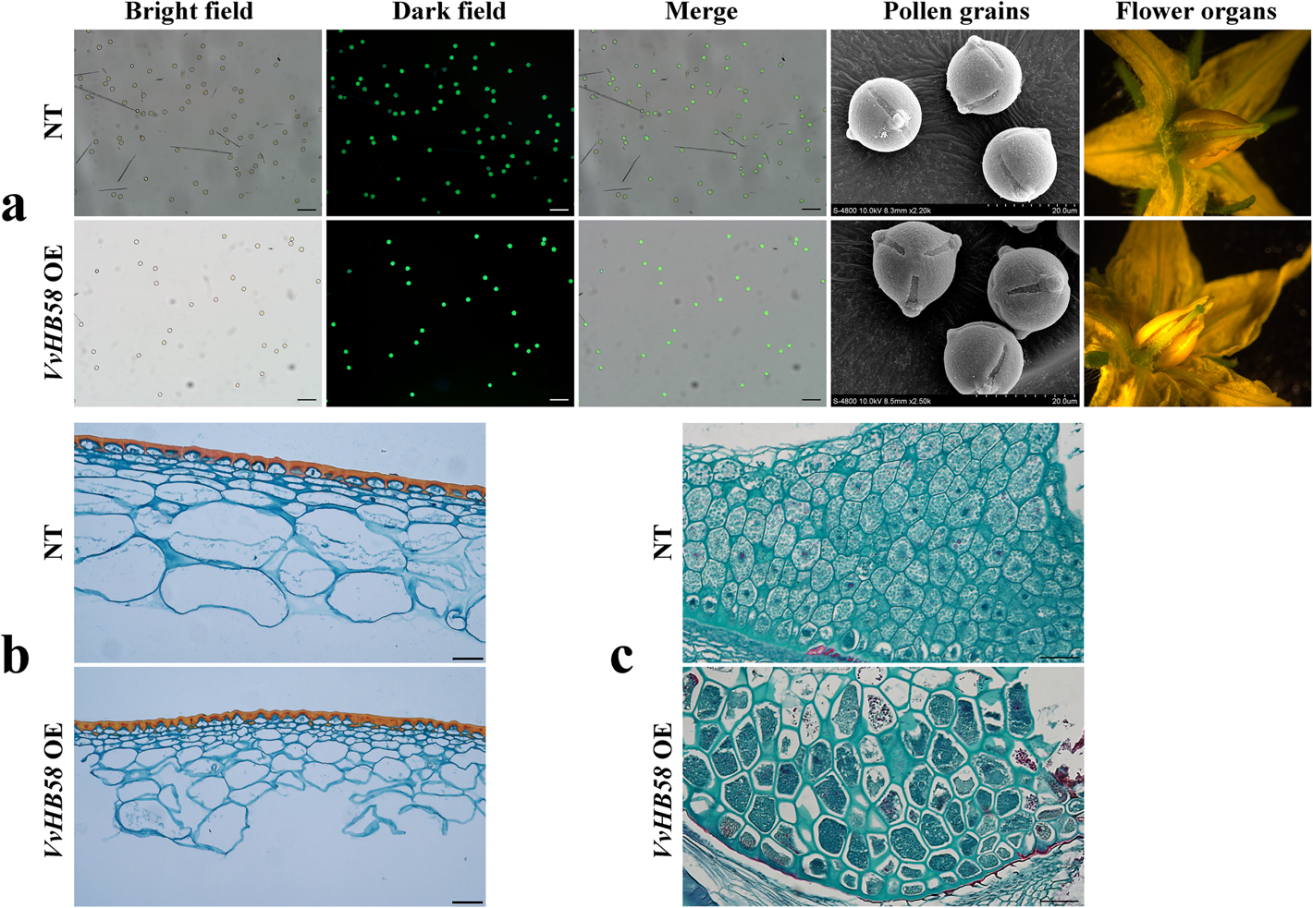
Morphologic observation of flowers, fruit and seeds of nontransgenic control plants and *VvHB58* overexpressing plants. **a** Determination of pollen viability and observation of pollen grain and flower morphology. Pollen viability was assessed by FDA staining. NT represents nontransgenic tomatoes. Bars, 100 μm. The morphology of pollen grains was observed using a scanning electron microscope (SEM). Bars, 20 μm. Flowers used in these experiments were collected at full bloom stage. **b** Morphological change in pericarp due to overexpression of *VvHB58*. Microscopic cross section through a pericarp of a control fruit and a *VvHB58* overexpressing progeny (T1) fruit from representative transformant line OE-8 was used. Bars, 50 μm. **c** Observation of longitudinal section in seeds between *VvHB58* overexpressing progeny and nontransgenic type. Bars, 50 μm

Subsequently, we studied the floral organs and pollen grains of transgenic tomatoes. As evaluated by FDA staining, pollen viability was not significantly affected by *VvHB58* expression (Fig. 4a). Additionally, we observed no difference in the size and shape of their pollen grains and floral organs (Fig. 4a). This suggests that *VvHB58* gene regulates fruit and seed development may not via affecting stamens or pistils. Furthermore, we observed that the pericarp of the fruit from *VvHB58* transgenic tomatoes was relatively thick compared with the control fruit. The pericarp cells of transgenic fruit (Fig. 4b) were smaller than controls, but the number of layers with small cells below the epidermis was increased, suggesting an additional role for *VvHB58* in fruit enlargement prior to ripening. Histological longitudinal section of seeds of transgenic and control plants showed that the endosperm cells in transgenic seeds were larger and more loosely arranged, suggesting that the increased seed size may be at least partly due to larger endosperm cells (Fig. 4c).

### Variations in phytohormone levels and hormone-related gene expression between *VvHB58* transgenic and control plants

**Fig. 5:**
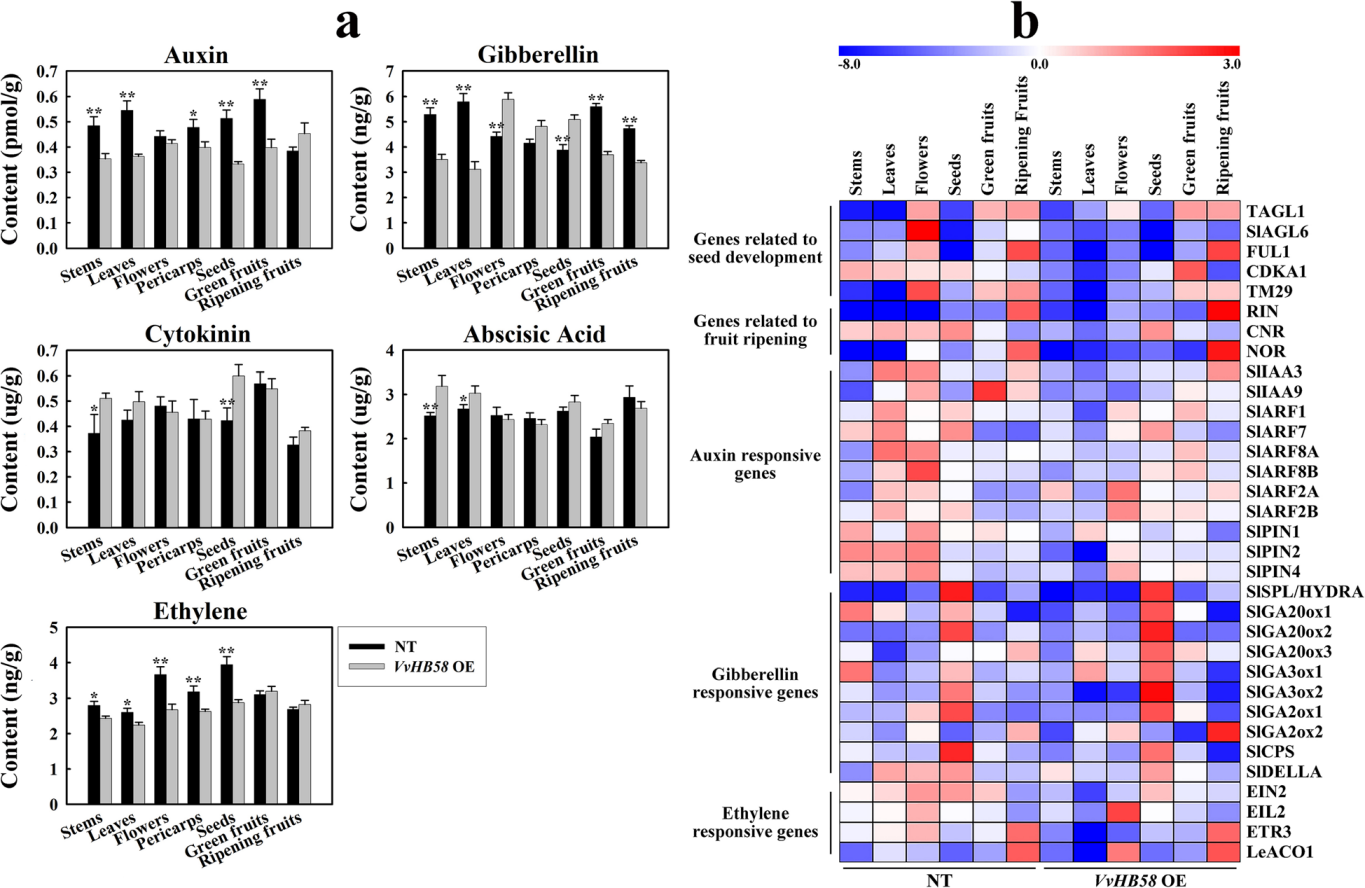
Changes in content of auxin, gibberellin, cytokinin, abscisic acid and ethylene, and expression patterns of hormone-related genes associated with expression of *VvHB58*. **a** Changes in content of endogenous auxin, gibberellin, ethylene, cytokinin and abscisic acid in nontransgenic tomatoes (NT) and transgenic tomatoes. **b** Expression analysis of auxin, gibberellin and ethylene responsive genes, seed development-related genes, and fruit ripening-related genes using qPCR. Transcript levels of each gene were normalized based on the expression of the *SlACTIN* gene. The resulting expression value of each gene was normalized on the basis of the mean expression value in all tissues, then was log2-transformed to form heat-maps through MultiExperiment Viewer 4.9.0 software. Red and blue color scale indicates high and low expression levels, respectively. Accession numbers for all genes are listed in Additional file 11: Table S3. Analysis of significance differences is shown in Additional file 2: Fig. S1 and Additional file 3: Fig. S2

Seed development process in tomato is strongly tied to endogenous concentrations of auxin and gibberellin. High levels of these two hormones in the ovary play an important role in fruit set and early development [36]. To explore whether the observed effect of *VvHB58* on the transgenic tomato fruit and seed is due to its influence on hormone signaling pathways, we measured the endogenous levels of the five classic phytohormones auxin, gibberellin, cytokinin, abscisic acid and ethylene in stems, leaves, flowers, pericarp, seeds, green fruit and ripening fruit, in both control and transgenic lines (Fig. 5a). We found that, in general, the content of endogenous auxin, gibberellin and ethylene were significantly different between transgenic and control plants. In contrast, cytokinin and abscisic acid levels showed no significant difference.

Except for flowers and ripening fruit, the content of endogenous auxin in transgenic tomatoes was significantly lower than that of control plants, and this is consistent with the the dwarfed stature and smaller fruit size of the transgenic tomatoes. Additionally, expression levels of several auxin responsive genes (*SlIAA3*, *SlIAA9*, *SlARF1*, *SlARF7*, *SlARF8A*, *SlARF8B*, *SlARF2A* and *SlARF2B*) described to be involved in the control of fruit initiation [36–38], and auxin distribution genes (*SlPIN1*, *SlPIN2* and *SlPIN4*) in the *SlPIN* gene family known to be involved in fruit development were evaluated via qPCR [39, 40] (Fig. 5b and Additional file 2: Figure S1). In general, these genes showed stronger expression in control plants than in transgenic tomatoes. For example, the expression *SlIAA3*, *SlARF8A* and *SlARF8B* were higher in leaves and flowers of nontransgenic control plants compared with transgenic tomatoes. Likewise, *SlPIN2* and *SlPIN4,* encoding PIN-like auxin efflux carriers, were expressed more strongly in stems, leaves and flowers of control plants. However, the expression levels of some genes did not coincide with the difference seen for auxin content. For instance, *SlARF2A* and *SlARF2B* showed increased expression in flowers and green fruit of transgenic tomatoes. This indicates that the regulatory effect of the *VvHB58* gene on auxin may be carried out through a relatively complex network.

The content of endogenous gibberellin in stems, leaves, green fruit and ripening fruit of transgenic tomatoes was significantly lower than that of control plants, but was markedly higher in flowers, pericarps and seeds (Fig. 5a). This is consistent with the reduced gibberellin content in stems, leaves, green fruits and ripening fruit conditioning the dwarfing and smaller fruit size in transgenic tomatoes, and increased gibberellin content in flowers conditioning the observed decrease of seed number per fruit. Furthermore, the increase of gibberellin content in seeds of transgenic tomatoes may be connected with the larger seed size. The expression levels of genes involved in gibberellin biosynthesis, such as *SlGA20ox1*, *SlGA20ox2*, *SlGA20ox3*, *SlGA3ox1*, *SlGA3ox2*, *SlCPS* (copalyl diphosphate synthase) and *SlSPL/HYDRA* (SPOROCYTELESS/*HYDRA*) [36, 41], gibberellin inactivation *SlGA2ox1* and *SlGA2ox2* [42], and gibberellin response *SlDELLA* were also detected [43] (Fig. 5b and Additional file 2: Figure S1). Most of these genes showed stronger expression in seeds, and the five genes related to gibberellin biosynthesis showed higher transcript levels in seeds of transgenic tomatoes compared with control plants. In transgenic tomatoes, up-regulation of the gibberellin inactivation genes *SlGA2ox1* and *SlGA2ox2* in green and ripening fruit would be expected to reduce levels of bioactive gibberellin. In a word, the increased transcript levels of gibberellin biosynthesis genes and decreased transcript levels of gibberellin inactivation genes should increase bioactive gibberellin, which could promote premature ovary growth.

Tomatoes are climacteric fruit, and endogenous ethylene has a strong promotive effect on fruit ripening. In the *VvHB58* transgenic tomatoes, the content of endogenous ethylene in stems, leaves, flowers, pericarps and seeds was dramatically lower than in control plants (Fig. 5a). We analyzed the transcript levels for the ethylene biosynthesis genes *LeACO1* (1-aminocyclopropane-1-carboxylate oxidase 1), and ethylene receptor genes *EIN2* (ethylene signaling protein), *EIL2* (EIN3-like proteins) and *ETR*3 (NR, never-ripe) by qPCR [37] (Fig. 5b and Additional file 2: Figure S1). *EIN2* and *ETR3* exhibited similar transcript levels, showing lower expression in stems, leaves, flowers and green fruits of transgenic tomatoes compared with control plants. In contrast, *EIL2* and *LeACO1* showed relatively higher transcript levels in flowers of transgenic tomatoes. Altogether, considering both ethylene content changes and gene expression patterns, we concluded that ethylene responses are slightly impaired in the transgenic tomatoes.

Fruit and seed development is a complex yet tightly regulated process. Except for auxin, gibberellin and ethylene, it is also regulated by abscisic acid and cytokinin. The increased endogenous cytokinin content in transgenic seeds may lead to seed enlargement (Fig. 5a). More abscisic acid in the stems and leaves of transgenic tomatoes may promote premature senescence (Fig. 5a). On the whole, the regulatory effect of *VvHB58* gene on fruits and seeds may be carried out mainly through changing endogenous auxin, gibberellin and ethylene content.

Additionally, we analyzed the expression levels of the fruit ripening-associated genes *RIN* (ripening inhibitor), *CNR* (colorless non-ripening) and *NOR* (nonripening) [37], and seed development-related gene *TAGL1* (*AGAMOUS-LIKE1*), *SlAGL6* (*SlAGAMOUS-LIKE 6*), *FUL1* (*FRUITFULL*, previously called TDR4), *CDKA1* (cyclin-dependent kinases) and *TM29* (Tomato *MADS-box 29*) [44–48] (Fig. 5b and Additional file 3: Figure S2). In transgenic tomatoes, expression of *NOR* and *RIN* was strongly reduced compared with controls plants in green fruit, but was enhanced in ripening fruits. This indicates that ectopic expression of *VvHB58* may cause a slight change in the fruit ripening mechanism. It has been reported that down-regulation of two genes, *TM29* and *SlAGL6*, can lead to parthenocarpy in tomato [45, 48]. Consistent with our results, the transcript levels of these two genes were dramatically lower in the flowers and fruits of transgenic tomatoes compared with control plants. Parthenocarpy in tomato can also be caused by up-regulation of the *CDKA1* gene [47]. Up-regulation of *CDKA1* during early fruit development, concomitant with rapid cell division, may also lead to parthenocarpy. We found that the *CDKA1* gene also exhibited higher transcript levels in green fruits of transgenic tomatoes. Furthermore, the *TAGL1* gene involved in carpel and stamen development was down-regulated in the flowers of transgenic tomatoes [49], and *FUL1* involved in fruit ripening exhibited a lower transcript level in flowers and green fruits of transgenic tomatoes. Taken together, this data suggests that *VvHB58* may affect seed and fruit development-related genes to co-regulate growth and development process of seed and fruit.

### Analysis of DNA methylation level in the *VvHB58* promoter

In order to elucidate the molecular basis of *VvHB58* differential expression, we cloned the *VvHB58* promoter from seeds DNA of ‘Thompson Seedless’ and ‘Red Globe’, including 1605 bp upstream from the ATG. We found that this sequence was invariant between the two cultivars (Additional file 4: Figure S3). Analysis of this region for cis-acting elements identified several elements implicated in hormone responses, defense and stress responses, light responses and endosperm expression (Additional file 4: Figure S3). Common among these were the GCN4 motif and Skn-1 motif related to endosperm expression, TGACG motif and CGTCA motif involved in methyl jasmonate responses, TCA element involved in salicylic acid responses, ABRE element associated with abscisic acid, HSE element involved in heat stress, MBS element related to drought inducibility, TC-rich repeats associated with defense and stress responses, LAMP element and AE-box involved in light response, and 5’ UTR Py-rich stretch conferring high transcription levels [27, 50]. The presence of these cis-acting elements suggests that *VvHB58* gene expression may be regulated by these different biological pathways.

**Fig. 6:**
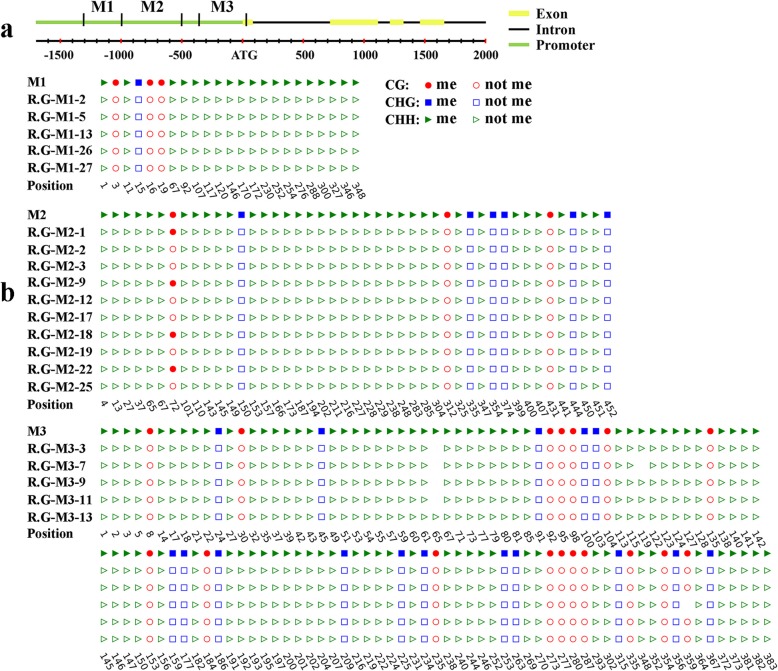
Analysis of DNA methylation in the *VvHB58* gene using bisulfite sequencing. **a** Structure of *VvHB58* gene. Three representative regions (M1, M2 and M3) were selected to detect DNA methylation level in ‘Red Globe’ (R.G). **b** Schematic diagram of bisulfite sequencing results. Red, blue, and green represent three types of cytosine methylation: symmetric CG, symmetric CHG, and asymmetric CHH (H = A, C, or T) respectively. Representative sequencing results of 5 or 10 single colonies were shown

To investigate if DNA methylation might be involved in the differential expression of *VvHB58* between seedless and seeded cultivars, bisulfite sequencing analysis was performed targeting three representative regions of the *VvHB58* gene in ‘Red Globe’ (Fig. 6a). M1 (-1309 bp ~ -898 bp) and M2 (-955 bp ~ -499 bp) were located in the promoter, and M3 (-360 bp ~ 28 bp) was located in the promoter and first exon region. As shown in Fig. 6b, the DNA methylation level of the *VvHB58* gene was very low, with only 40% CG type methylation in one CG context (-884 bp) of the M2 region. However, we found no cis-acting element near this CG site (Additional file 4: Figure S3), suggesting that lower expression of *VvHB58* in ‘Red Globe’ grape is independent of DNA methylation, and suggesting that other mechanisms may be responsible. However, it is also possible that regulatory changes in DNA methylation occurred outside of this identified region.

### Expression patterns of genes encoding DNA methyltransferases and demethylases in seedless and seeded grape

**Fig. 7:**
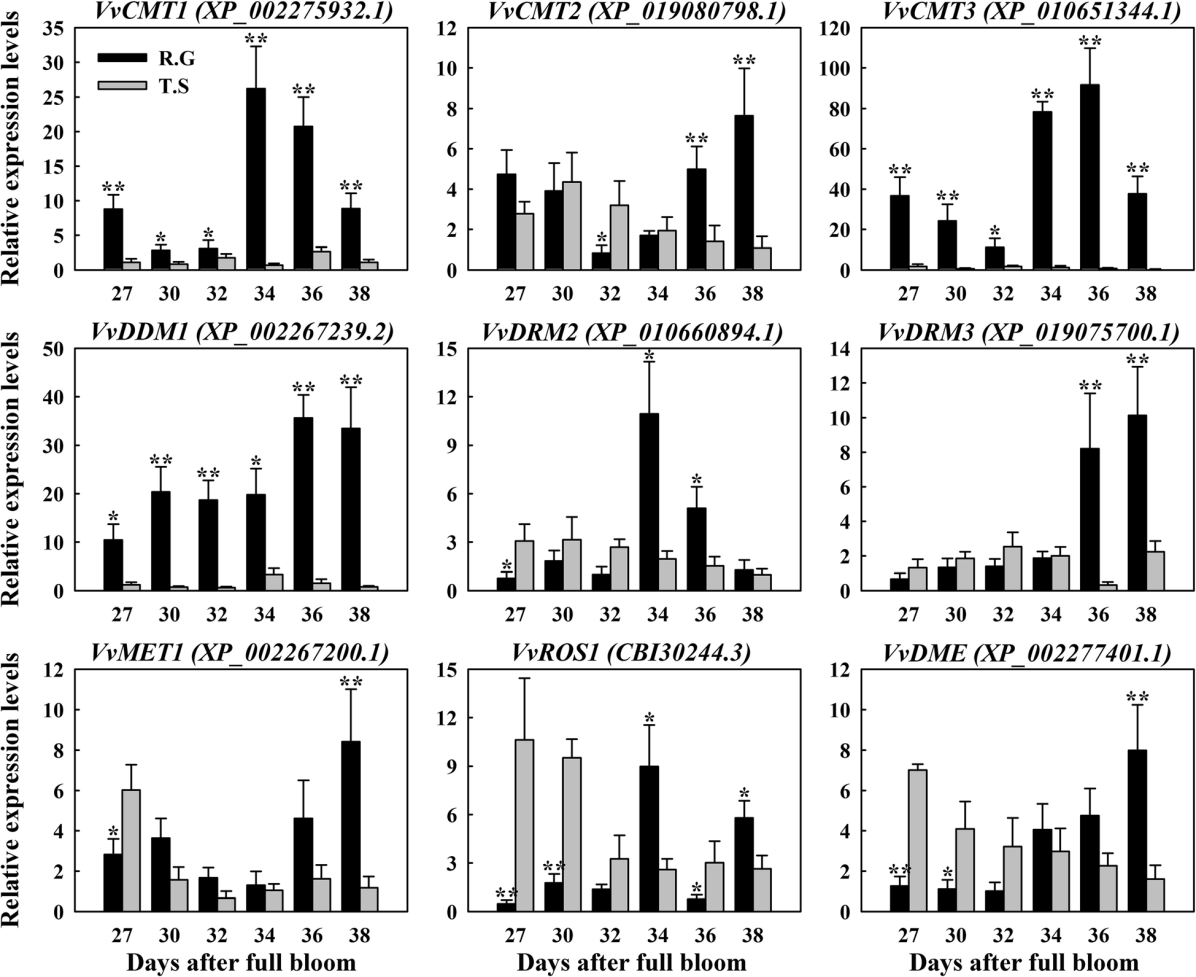
Relative expression analysis of genes encoding homologs of DNA methyltransferases and demethylases during different seed developmental stages using qPCR. ‘Red Globe’ and ‘Thompson Seedless’ are denoted as ‘R.G’ and ‘T.S’ respectively. Transcripts were normalized to the expression levels of the grapevine *Actin* and *GAPDH* genes. Bars represent means ± SD from three biological replicates. Significant differences were calculated from three biological replicates using one-way ANOVA are indicated by * (0.01 < *P* value < 0.05) and ** (*P* value < 0.01)

Cytosine methyltransferases and demethylases play vital roles in maintenance of genomic methylation and are involved in various biological processes, such as gametogenesis [16, 18], apomixis [51] and fruit ripening [21]. To identify potential methyltransferases and demethylases in the grape genome scale, we carried out a homology-based search for sequences encoding grapevine cytosine methyltransferases and demethylases, using the NCBI-blastp (https://blast.ncbi.nlm.nih.gov/Blast.cgi) and using characterized methylation factors from Arabidopsis as queries. We identified and characterized the expression during seed development of methyltransferases *VvCMT1* (XP_002275932.1), *VvCMT2* (XP_019080798.1), *VvCMT3* (XP_010651344.1), *VvDDM1* (XP_002267239.2), *VvDRM2* (XP_010660894.1), *VvDRM3* (XP_019075700.1) and *VvMET1* (XP_002267200.1), and demethylases *VvDME* (XP_002277401.1) and *VvROS1* (CBI30244.3) (Fig. 7 and Additional file 5: Table S2).

In the period from 27 to 38 DAF the expression of *VvCMT1*, *VvCMT3* and *VvDDM1* were significantly higher in ‘Red Globe’ than in ‘Thompson Seedless’. Likewise, in the period from 34 to 38 DAF the expression levels of *VvCMT2*, *VvDRM2*, *VvDRM3* and *VvMET1* were higher in ‘Red Globe’ than ‘Thompson Seedless’. In contrast, the expression of *VvROS1* and *VvDME* in ‘Thompson Seedless’ were higher than in ‘Red Globe’ during 27–32 DAF, then gradually declined during 34–38 DAF. In general, their expression levels gradually declined in ‘Thompson Seedless’ and gradually increased in ‘Red Globe’. The observation that grape seed development is related to changes in transcriptional levels of DNA methylation-related genes suggests that changes in DNA methylation may play a potential role in grape seed development.

## Discussion

### *VvHB58* encodes a HD-zip I protein involved in seed and fruit development

VvHB58 shares strong sequence similarity with Arabidopsis ATHB1 and tomato LeHB1 (Fig. 2). AtHB1 is involved in leaf development and hypocotyl elongation [35]. LeHB1 is not only involved in regulating fruit ripening via interacting with the promoter of *LeACO1*, but also plays a vital role in floral organogenesis [23]. Additionally, a homologous protein in apple, designated MdHB1, acts as a repressor of anthocyanin biosynthesis [52]. Taken together with the observation that *VvHB58* is expressed in many tissues and organs in grapevine, we predict that *VvHB58* possesses multiple functions during grapevine growth and development. This is consistent with previous observations that other HD-ZIP family members have diverse functions in plant development, especially embryonic development and hormone response, like apical embryo patterning, embryonic shoot meristem formation, and auxin response [22]. Interestingly, the HD-Zip subfamily seems to be specific to plants [28].

Combining previous transcriptional analysis of seed development in grape hybrids [11] and transcript levels of the *VvHB58* gene documented in this study (Fig. 1a, b), we found that *VvHB58* is expressed differentially during seed development between seedless and seeded grapes. The relatively strong expression of *VvHB58* in seeds of seedless cultivars, relative to seeded cultivars, suggests that *VvHB58* participates in seed development. Previous transcriptome analyses of seed development in grape hybrids also indicated that homeobox transcription factors may regulate seed development-related processes [1, 11].

### *VvHB58* influences seed and fruit development through auxin, gibberellin and ethylene signaling pathways

We further analysed the potential function of *VvHB58* via heterologous expression in tomato. From the perspective of expression levels, *VvHB58* is a differentially expressed gene during seed development stages in seedless and seeded grapes, showing a higher expression level in seeds of seedless grape. It is speculated that *VvHB58* may affect seed development in seedless grapes, and further affect the fruit development. In *VvHB58* transgenic tomatoes, *VvHB58* tend to be expressed in the transgenic tomato seeds (Fig. 3h), suggesting *VvHB58* may be a gene specifically expressed in seeds. *VvHB58* regulates the transcript levels of key genes in the auxin, gibberellin, cytokinin and ethylene signaling pathways in seeds, thereby further regulates the hormone network in fruits to affect the fruit development process. By comparing the nontransgenic control and *VvHB58* transgenic fruit, we concluded that *VvHB58* impacts pericarp cell expansion, fruit size and seed size and number, and that this was related to changes in contents of endogenous auxin, gibberellin and ethylene. Therefore, we hypothesize that the effect of *VvHB58* is mediated through these hormone signaling pathways.

The expression level of *LeHB1* gene (a *VvHB58* homologous gene in tomatoes) increased only in green fruit stage in *VvHB58* transgenic tomatoes. The expression patterns and functions of the two genes were different. *LeHB1* can regulate the promoter of *LeACO1* to further regulate fruit ripening. Inhibition of *LeHB1* mRNA accumulation in tomato fruit greatly reduced *LeACO1* mRNA levels, and inhibited ripening, and overexpression of *LeHB1* altered floral organ morphology [23]. In our study, *VvHB58* transgenic tomatoes did not exhibit these phenotypes so we speculated that the phenotype of *VvHB58* transgenic tomatoes was not caused by *LeHB1*.

**Fig. 8:**
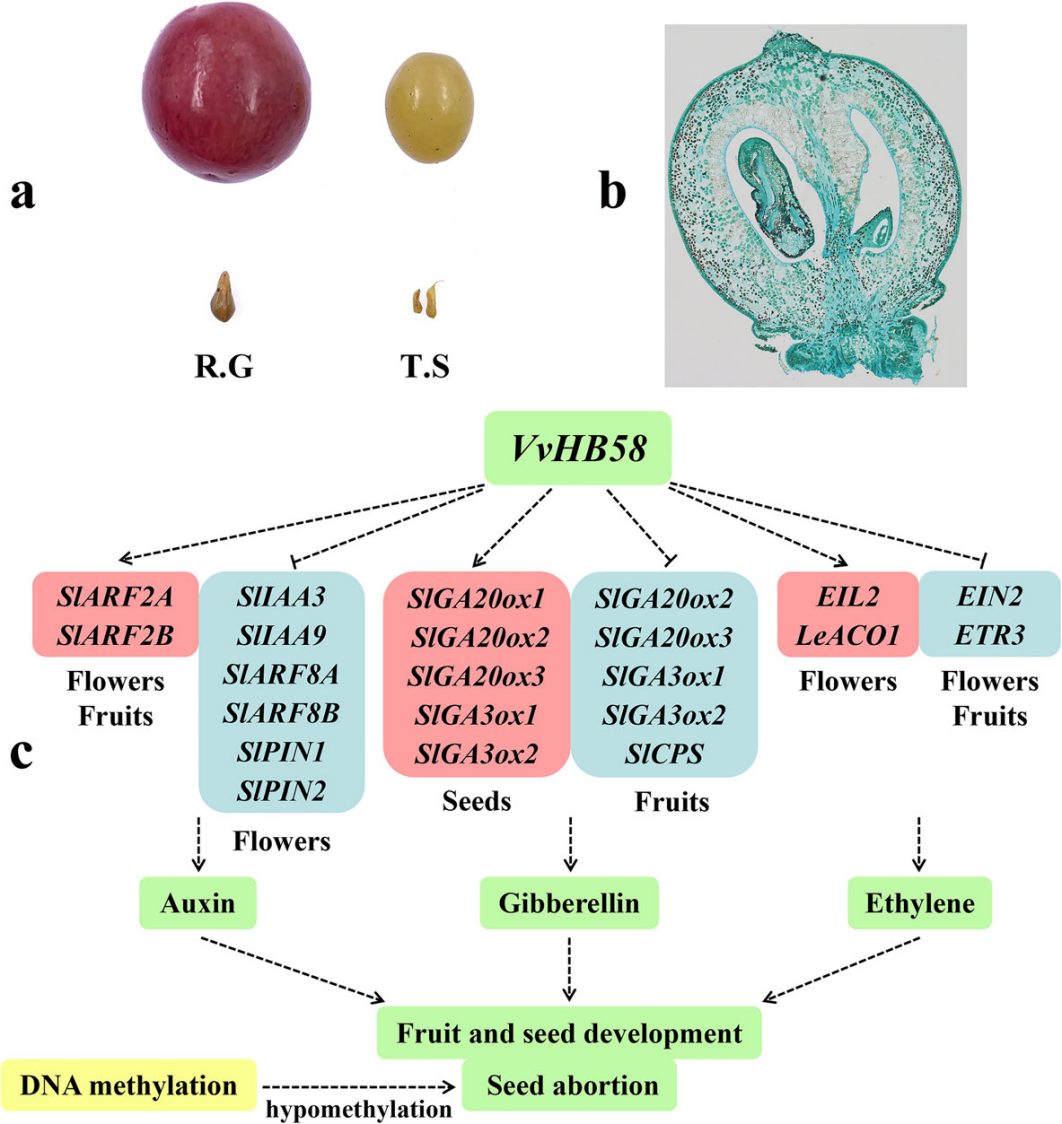
Morphological observation of seedless and seeded grapes, and a hypothetical model for *VvHB58* in regulating fruit and seed development. **a** Phenotypic observation of seedless ‘Thompson Seedless’ (T.S) and seeded ‘Red Globe’ (R.G) grape. **b** Morphologic observation of fruit from ‘Thompson Seedless’. **c** A hypothetical model for regulatory mechanisms involving the *VvHB58* gene

Grape fruit development involves a complicated interaction of physiological and biochemical processes [13]. The first stage begins after pollination, at fruit set. During 2 weeks after flowering, auxin and gibberellin directly promote rapid cell division and enlargement to increase the berry size exponentially, simultaneously leading to the establishment of an eventual cell number. Micro-Tom is a useful system to investigate fruit set and development. Because Micro-Tom has a mutation in the *SELF-PRUNING* (SP) and *DWARF* (D) genes, the response of Micro-Tom to gibberellin is unbalanced. The dwarf habit of Micro-Tom might be related to an altered GA response. In our study, compared with non-transgenic tomatoes, *VvHB58* transgenic tomatoes were obviously dwarfed. The gibberellin levels in stems and leaves of *VvHB58* transgenic tomatoes were significantly decreased, and the transcription levels of key genes in gibberellin pathway were also changed. This suggested that the dwarfing of transgenic tomatoes may be related to the change of GA pathway [53]. Additionally, auxin-related genes were expressed differentially in flowers and fruit of *VvHB58* transgenic tomatoes relative to control plants, and gibberellin-related genes were expressed differentially in seeds and fruits. Therefore, we speculate that *VvHB58* may down-regulated auxin and gibberellin signaling in transgenic fruit, thus reducing fruit size (Fig. 5a and Fig. 8c).

‘Red Globe’ fruit typically contain 2–4 seeds, whereas ‘Thompson Seedless’ contains only 1–2 small and hollow seeds and 1–2 seeds with totally aborted embryo and endosperm (Fig. 8a, b). In general, compared with seeded grapes, the fruit size and seed size of seedless grapes is smaller, and seeds are softer and generally lack seed coat lignification and normally developing embryo and endosperm. This phenotypic difference involves many biological pathways, and many key genes related to grape seedlessness have also been identified [1, 11]. In this study, we reported a *VvHB58* gene, which may play a potential role in regulating seed number and berry size. Our observation that the gibberellin content in seeds of *VvHB58* transgenic tomatoes was higher than control plants is consistent with a previous study showing that endogenesis gibberellin was higher in seedless grapevine progeny than seeded progeny during seed development [11]. Additionally, exogenous application of gibberellin to grapevine flowers prior to bloom can result in seed abortion [12], and high levels of endogenous gibberellin in tomato flowers is related to parthenocarpy [36]. We assume that the remarkable increase in gibberellin in *VvHB58* transgenic flowers may be related to the decrease of seed number (Fig. 5a).

Ethylene is also involved in development and ripening of climacteric fruits. SlTPR1 (a tomato tetratricopeptide repeat protein) can regulate the transcript levels of *ChitB* (an ethylene responsive gene), *SlSAUR1-like* (an auxin responsive gene) and *SlIAA9* to cause ethylene-related pleiotropic effects including infertility and parthenocarpy [54]. Remarkable changes in ethylene content and transcript levels of ethylene-related genes between *VvHB58* transgenic and control plants also suggest that ethylene plays a vital role in fruit and seed development. Taken together, we suspect seed and fruit development may be controlled by the synergistic regulation of multiple hormones.

### The differential expression pattern of *VvHB58* between seedless and seeded cultivars may be caused by different transcriptional regulatory mechanisms rather than DNA methylation of the promoter

We found that the ~ 1605-base promoter sequence of *VvHB58* was invariant between ‘Thompson Seedless’ and ‘Red Globe’, indicating that the differential expression of this gene during seed development in the two cultivars is mediated by sequence elements outside this region, or by cultivar-specific trans-acting mechanisms. We found multiple cis-acting elements involved in hormone responses and endosperm expression in this 1605 bp region (Additional file 4: Figure S3). Differences in the activity of these cis-acting elements in seedless and seeded cultivars may lead to differential expression of *VvHB58* gene. In addition to different transcriptional regulation mechanisms, DNA methylation can also regulate gene expression without changing DNA sequence. Through bisulfite sequencing, we found that the cytosine methylation levels across the *VvHB58* gene were extremely low in the seeds of ‘Red Globe’ (Fig. 6). So, the extremely low expression level of *VvHB58* in ‘Red Globe’ is not due to DNA methylation inhibition. Because the M3 regions includes the most potential methylated cytosine contexts (CG, CHG and CHH, where H is A, C or T), we further analyzed the DNA methylation level of M3 region in seedless grape ‘Thompson Seedless’. As shown in Additional file 6: Figure S4, cytosine methylation was also low in ‘Thompson Seedless’. Therefore, it can be concluded that the differential transcript levels of *VvHB58* gene between seedless and seeded grapes was not associated with DNA methylation in this region. However, there is the possibility that differential methylation of a few bases not detected here may have a regulatory function.

### DNA methylation may play a vital role in seed development process in seedless grapes

In plant, DNA can be methylated at cytosine bases in all sequence contexts CG, CHG and CHH, where H represents A, T or C [17]. The establishment, maintenance and removal of DNA methylation involve different mechanisms. De novo DNA methylation is mediated via the RdDM pathway (RNA-directed DNA methylation), which also includes siRNAs (small interfering RNAs), scaffold RNAs and an array of proteins [17]. The RdDM pathway is responsible for methylation at asymmetric CHH sites through two methyltransferases DRM1 and DRM2 (Domains Rearranged Methylases). Maintenance of DNA methylation at symmetrical CG sites is catalysed by Methyltransferase1 (MET1), whereas CHG methylation is maintained by CMT2 or CMT3. CHH methylation is maintained through DRM2 or CMT2. Additionally, CHG methylation and histone H3K9me2 methylation can reinforce each other via feedback regulation [15]. Cytosine methylation maintenance for symmetric sequence contexts also requires a chromatin remodelling protein, DDM1 (Decresed DNA Methylation 1). CMT2-mediated methylation is affected by mutations in DDM1 [15].

Additionally, following DNA replication, the lack of DNA methyltransferase activity or a methyl donor leads to passive DNA demethylation. DNA methylation can also be erased via a team of bifunctional 5-mC DNA glycosylases, including ROS1 (REPRESSOR OF SILENCING 1), DME2 (TRANSCRIPTIONAL ACTIVATOR DEMETER), DML2 (DEMETER-LIKE PROTEIN 2) and DML3.

DNA methylation possesses broad-ranging functions in regulating gene expression, chromosome interactions, transposon silencing and trait inheritance [15]. At present, analyses of genomic DNA methylation in seeds has been restricted to a few model plants [55]. Increasing evidence suggests that DNA methylation plays a vital role in regulating seed and endosperm development and seed size [14, 56]. For example, as a major regulator of gene imprinting, DNA hypomethylation in Arabidopsis has a parental effect in regulating seed size [14]. In castor bean (*Ricinus communis*), it has been demonstrated that siRNAs (typically 24 nucleotide) play a vital role in maintenance of CHH methylation and inhibit the activation of TEs (transposable element) in persistent endosperm development [55].

In this study, we found that specific DNA methylation-related genes exhibited differential expression during seed development between seedless and seeded grapes (Fig. 7). We hypothesize that the establishment, maintenance and removal mechanisms of DNA methylation are different in seedless and seeded grapes, especially involving VvCMT1, VvCMT3 and DDM1-mediated methylation pathways. On the whole, DNA methyltransferase-related genes (*VvCMT1*, *VvCMT2*, *VvCMT3*, *VvDDM1*, *VvDRM2*, *VvDRM3* and *VvMET1*) showed higher transcript levels in ‘Red Globe’ than ‘Thompson Seedless’. The transcript levels of DNA demethylase-related genes (*VvROS1* and *VvDME*) were dramatically lower during 27–32 DAF in ‘Red Globe’. This suggests that normal seed development requires relatively higher genomic DNA methylation levels, and hypomethylation may be associated with seed abortion during seed development (Fig. 8c). This hypothesis needs to be further confirmed by methylome analysis in seedless and seeded grapes.

## Conclusion

In this study, we used the breadth of genetic and molecular tools to investigate possible functional and regulatory mechanisms involving *VvHB58* during seed development. *VvHB58* has a potential function in regulating fruit size and seed size and number through affecting multiple hormonal pathways. Additionally, the markedly lower DNA methylation levels during seed abortion in seedless grapes suggested that hypomethylation might be related to seed abortion. Overall, these results provide insights into possible regulatory mechanisms on fruit size and seed number, which should greatly facilitate further study and molecular breeding in grapes.

## Methods

### Plant materials

Five grapevine cultivars were used in this study: Two seedless cultivars, ‘Thompson Seedless’ (*Vitis vinifera*) and ‘Flame Seedless’ (*V. vinifera*), and three seeded cultivars, ‘Red Globe’ (*V. vinifera*), ‘Kyoho’ (*V. vinifera* × *V. labrusca*) and ‘Muscat Hamburg’ (*V. vinifera*). All plants were maintained in the grape germplasm resource orchard of Northwest Agriculture & Forestry University, Yangling, China (34°20′N 108°24′E). Seeds/seed traces at 27, 30, 33, 36, 39 and 42 days after full bloom (DAF) were collected from 3-year-old ‘Thompson seedless’, ‘Flame Seedless’, ‘Red Globe’ and ‘Kyoho’. Seeds/seed traces at 21, 24, 27, 30, 32, 34, 36, 38, 40, 42, 45 and 48 DAF were collected from 4-year-old ‘Thompson Seedless’ and ‘Red Globe’. Seeds/seed traces at 27, 30, 32, 34, 36 and 38 DAF were used for expression analysis of methylation-related genes. For analysis of developmental expression, roots, stems, leaves, tendrils and fruits (at 42 DAF), flowers (at full bloom stage), and floral organs were collected from 3-year-old ‘Flame Seedless’ and ‘Muscat Hamburg’. Samples were immediately frozen in liquid nitrogen and stored at -80 °C.

Micro-Tom seeds were bought from the PanAmerican Seed company (USA). Professor Qingmei Guan from Northwest Agriculture & Forestry University provided the tobacco (*Nicotiana benthamiana*) used in this study. The *VvHB58* transgenic tomatoes that were obtained for this work cultivation complies with China’s legislation on genetically modified plants.

### Phylogeny and sequence alignment

Phylogeny of predicted HD-Zip I proteins from grape (*Vitis vinifera*), tomato (*Solanum lycopersicum*), Arabidopsis (*Arabidopsis thaliana*), rice (*Oryza sativa*), soybean (*Glycine max*) and maize (*Zea mays*) was constructed by MEGA 6.0 software using neighbour-joining (NJ) method [57]. Full-length protein (predicted open reading frame translation) sequences were used, and bootstrap analysis was performed with 1000 replicates and p-distance. Multiple sequence alignment was performed by DNAMAN software with default parameters.

### qPCR analysis

Total RNA was extracted using the EZNA Plant RNA Kit (R6827-01, OMEGA Biotek, USA). The PrimeScript™ RT reagent Kit with gDNA Eraser (TaKaRa Biotechnology, Dalian, China) was used to remove gDNA and then for reverse transcription. Genome DNA was removed by gDNA Eraser (contains DNase). A qPCR reaction on crude RNA was also performed in order to access the degree of gDNA contamination. The quality of RNA was determined by agarose gel electrophoresis. The purity and concentration of RNA used for cDNA synthesis were also examined by spectrophotometry. The A260/A280 ratio is between 1.9 and 2.1. A total of 1 μg DNase-treated RNA was reverse-transcribed to cDNA. The cDNA products were diluted six-fold and stored at -40 °C for gene cloning and qPCR analysis. A qPCR reaction was performed on an IQ5 real time-PCR machine (Bio-Rad, Hercules, CA, USA) with SYBR® Premix Ex Taq™ II (TaKaRa). In order to detect the specificity of primers used in qPCR, we examined whether the melting curve of each gene presented a single peak (Additional files 7, 8, 9, and 10: Figure S5-8). The length and specificity of qPCR product were checked by agarose gel electrophoresis. Moreover, the PCR products were sequenced to clarify the validity and completeness. The two internal reference genes grape *Actin* (GenBank Accession number: AY680701) and *GAPDH* (GenBank Accession number: CB973647) were used to normalize target gene expression levels. Expression levels were normalized against the geometric mean of the two reference genes, using the Hellemans equation [58] in qbase+ software. Analysis of qPCR data was performed using the qbase+ (Biogazelle, Genth, Belgium) based on the delta-Cq quantification model as previously described [58]. Each assay was run with three independent biological replications. Primers and accession numbers of all genes used in qPCR analysis are listed in Additional file 11: Table S3.

### Subcellular localization assays

A DNA fragment corresponding to the coding sequence of *VvHB58* was amplified by PCR and then cloned into the pEarleyGate101 vector via Gateway recombination technology (Invitrogen) [59]. The resulting construct and empty pEarleyGate101 vector were transformed into *Agrobacterium tumefaciens* strain GV3101 through electroporation. The leaves of 5-week-old tobacco (*Nicotiana benthamiana*) plants grown in a growth chamber (16 h light/8 h dark, 22 °C/18 °C) were used for transient expression. The *A. tumefaciens* cultures were resuspended in infiltration buffer (10 mM MgCl2, 10 mM MES and 150 μM acetosyringone) at a final OD600 of 1.0. The infiltrated tobacco plants were grown for an additional 72 h prior to microscopic examination. Yellow fluorescent protein (YFP, excitation wavelength: 513 nm, emission wavelength: 527 nm) signals were observed with a Nikon A1R/A1 confocal microscope (Nikon, Tokyo, Japan). Blue fluorescent signals emitted by the 4,6-diamidino-2-phenylindole dihydrochloride (DAPI, excitation wavelength: 358 nm, emission wavelength: 461 nm) were used to identify the nucleus [59].

### Agrobacterium-mediated transformation of tomato

The full coding sequence of *VvHB58* amplified from Seeds of ‘Thompson Seedless’ was cloned into the pCAMBIA2300 vector to form CaMV35S:VvHB58. The resulting construct and empty pCAMBIA2300 vector were transferred into *A. tumefaciens* strain GV3101 through electroporation. *A. tumefaciens*-mediated transformation was performed using young cotyledons of the micro-Tom. Non-transgenic and transgenic tomatoes were grown in a growth chamber (16 h light/8 h dark, 22 °C/18 °C). Transgenic lines were verified with primers specific for the CaMV35S:VvHB58 construct using RT-PCR. The primers used for constructing vectors are shown in Additional file 12: Table S4. A total of eight T0 lines were obtained in our study. At least ten T1 plants were obtained from each line for observation. We randomly selected two lines (OE-3 and OE-8) for subsequent analysis. We also observed and counted the traits of another six lines. Their phenotypes and traits are consistent with OE-3 and OE-8 shown in the manuscript.

### Cytological and phenotypic analyses

Pollen viability was assessed via FDA staining [60]. Anthers were isolated under a stereoscope and transferred into FDA solution, then gently broken with a fine needle and carefully squeezed with a cover slip to release the pollen. Pollen morphology was observed through a scanning electron microscope (SEM) as described previously [60].

### Determination of plant hormone concentrations

The extraction, purification, and determination of endogenous auxin, abscisic acid, gibberellin (GA_1 + 3_ and GA_4 + 7_), ethylene (conjugated ethylene) and cytokinin were performed using an enzyme-linked immunosorbent assay (ELISA) as previously described [61]. The detection ranges of IAA, ABA, GAs, ETH and CTK are 2 pmol/L - 48 pmol/L, 10 μg/L - 300 μg/L, 20 pg/ml - 480 pg/ml, 10 ng/L - 320 ng/L and 1 μg/L - 48 μg/L respectively. Samples consisting approximately 0.4 g plant tissue were ground in liquid nitrogen, and hormones were extracted overnight at 4 °C by cold 80% (v/v) methanol containing 1 mM butylated hydroxytoluence as an antioxidant. The extracts were collected after centrifugation at 4 °C for 20 min (10,000×g) and passed through a C_18_ Sep-Pak cartridge (Waters, Milford, MA, USA), then dried under N_2_. The residues were dissolved in 10 mM phosphate buffer saline (PBS) (pH 7.4) to determine the levels of IAA, ABA, GAs (GA_1 + 3_ and GA_4 + 7_), conjugated ETH and CTK. ELISA was performed on a 96-well Micro-titration plate. Each well was coated with 50 mM NaHCO_3_ buffer (pH 9.6) containing synthetic IAA, ABA, GAs (GA_1 + 3_ and GA_4 + 7_), ETH and CTK -ovalbumin conjugates. After overnight at 37 °C, the ovalbumin solution was added to each well to block non-specific binding. Then, standard IAA, ABA, GAs (GA_1 + 3_ and GA_4 + 7_), ETH and CTK samples, and antibodies, were added. They were incubated at 37 °C for 45 min. The antibodies against each hormone were monoclonal antibodies obtained as previously described [62]. Because GA_1_ and GA_3_ or GA_4_ and GA_7_ are too similar to be separated from each other, only two different antibodies were used for GA_1 + 3_ and GA_4 + 7_. The horseradish peroxidase-labeled goat anti-rabbit immunoglobulin was added to each well and incubated at 37 °C for 60 min. Then, the buffered enzyme substrate (ortho-phenylenediamine) was added, and the reaction was carried out in darkness at 37 C for 15 min, then terminated with 3 M H_2_SO_4_. The absorbance at 490 nm was recorded using an ELISA spectrophotometer. Calculations of the enzyme-linked immunosorbent data were performed as previously described [62]. The results are the means ± SE of at least three biological replicates.

### Promoter *cis*-element analysis

*VvHB58* promoter sequence was obtained from the Grape Genome Database (12×; http://www.genoscope.cns.fr). A 1605 bp promoter segment was cloned from DNA derived from seeds of ‘Thompson seedless’ and ‘Red Globe’, and cis-acting regulatory elements were predicted using the online program PlantCARE (http://bioinformatics.psb.ugent.be/webtools/plantcare/html/).

### Analysis of DNA methylation within the *VvHB58* gene

Genomic DNA was extracted from seeds of ‘Red Globe’ and ‘Thompson Seedless’ using a CTAB-based method, and treated with bisulfite using the EZ DNA Methylation-Gold kit (D5005, Zymo Research, USA) as done previously [63]. PCR products were amplified from the bisulfite-modified DNA with primers (Additional file 13: Table S5) targeting three regions (M1, M2 and M3) located within the promoter and first exon of the *VvHB58* gene. PCR products were purified and cloned into the pGEM-T Easy vector (Promega). At least 20 single colonies of each clone were sequenced. The methylation level of the cloned sequences was analyzed using the online tool CyMATE (http://www.cymate.org/) [64].

### Statistical analysis

Data analysis was carried out using Microsoft Excel (Microsoft Corporation, USA) and data were plotted using Sigmaplot 12.0. One-way ANOVA was performed to assess significant differences using the SPSS Statistics 22.0 software.**Notes.**

Yunduan Li and Songlin Zhang contributed equally to this work.

## Supplementary information

**Additional file 1: Table S1.** Accession numbers of HD-Zip I proteins in *Arabidopsis thaliana*, *Solanum lycopersicum*, *Vitis vinifera*, *Oryza sativa*, *Zea mays* and *Glycine max*.

**Additional file 2: Figure S1.** Transcript levels of auxin, gibberellin and ethylene responsive genes between *VvHB58* transgenic and nontransgenic tomatoes using qPCR. Transcripts of SlACTIN gene were used as an endogenous control. NT represents nontransgenic tomatoes. Bars represent means ± SD from three biological replicates. Asterisks stand for statistical significance (*0.01 < *P* < 0.05, **P < 0.01, one-way ANOVA).

**Additional file 3: Figure S2.** Expression analysis of seed development-related genes, and fruit ripening-related genes between VvHB58 transgenic and nontransgenic tomatoes via qPCR. Data were normalized to the expression levels of *Solanum lycopersicum SlACTIN* gene. NT represents nontransgenic tomatoes. Each value represents the means ± SD of three independent biological replicates. Significant differences were indicated by *0.01 < *P* < 0.05, **P < 0.01, one-way ANOVA.

**Additional file 4: Figure S3.** Analysis of cis-acting elements of *VvHB58* promoter and alignment of promoter sequences obtained from seedless grape (Thompson Seedless, T.S) and seeded grape (Red Globe, R.G).

**Additional file 5: Table S2.** Homologs of DNA methyltransferase and demethylase proteins in grape.

**Additional file 6: Figure S4.** Analysis of DNA methylation in M3 region of *VvHB58* gene in ‘Thompson Seedless’ grape using bisulfite sequencing. Red, blue, and green stand for three types of cytosine methylation symmetric CG, symmetric CHG, and asymmetric CHH (H = A, C, or T) respectively. Representative sequencing results of 5 single colonies were shown.

**Additional file 7: Figure S5.** The melting curve of each gene used in qPCR. The melting curve of each gene was a single peak. In the amplification plot, the purple line represents the negative control and the green line represents the sample analysis.

**Additional file 8: Figure S6.** The melting curve of each gene used in qPCR.

**Additional file 9: Figure S7.** The melting curve of each gene used in qPCR.

**Additional file 10: Figure S8.** The melting curve of each gene used in qPCR.

**Additional file 11: Table S3.** Primers used for qPCR. In the qPCR reaction, the amplification temperature of these genes were 60 °C. SGN: Sol Genomics Network (https://solgenomics.net/).

**Additional file 12: Table S4.** Primers used for vector construction.

**Additional file 13: Table S5.** Primers used for detection of DNA methylation levels.

## Data Availability

The datasets supporting the conclusions of this article are included within the article and additional files.

## Abbreviations

CNR: Colorless non-ripening
DAF: Days after full bloom
DRM: Domains Rearranged Methylases
HB: Homeobox
MET: Methyltransferase
NOR: Non-ripening
RdDM: RNA-directed DNA methylation
RIN: Ripening inhibitor
TEs: Transposable element
TFs: Transcription factors
