# Supplementary material for: The grapevine homeobox gene VvHB58 influences seed and fruit development through multiple hormonal signaling pathways
Source: BMC Plant Biol. 2019 Nov 27;19:523. doi: 10.1186/s12870-019-2144-9 (PMC6882351; doi:10.1186/s12870-019-2144-9)
Supplement: Supplementary file 1 — Additional file 1: Table S1. Accession numbers of HD-Zip I proteins in Arabidopsis thaliana, Solanum lycopersicum, Vitis vinifera, Oryza sativa, Zea mays and Glycine max. [file 12870_2019_2144_MOESM1_ESM.doc]

Additional file 1: Table S1. Accession numbers of HD-ZiP I proteins in Arabidopsis thaliana, Solanum lycopersicum, Vitis vinifera, Oryza sativa, Zea mays and Glycine max.

| ***Arabidopsis thaliana*** | | ***Solanum lycopersicum*** | | ***Vitis vinifera*** | | ***Oryza sativa*** | | ***Zea mays*** | | ***Glycine max*** | |
| --- | --- | --- | --- | --- | --- | --- | --- | --- | --- | --- | --- |
| **Gene ID** | **Gene locus ID** | **Gene ID** | **Gene locus ID** | **Gene ID** | **Gene locus ID** | **Gene ID** | **Gene locus ID** | **Gene ID** | **Gene locus ID** | **Gene ID** | **Gene locus ID** |
| ATHB1 | AT3G01470 | SLZH01 | Solyc01g010600 | VvHB04 | GSVIVT01020078001 | Oshox4 | Os09g29460 | Zmhdz1 | GRMZM2G021339 | Gmhdz2 | Glyma01g04890 |
| ATHB3 | AT5G15150 | SLZH05 | Solyc01g096320 | VvHB05 | GSVIVT01020033001 | Oshox5 | Os08g32080 | Zmhdz2 | GRMZM2G122076 | Gmhdz3 | Glyma01g05230 |
| ATHB5 | AT5G65310 | SLZH08 | Solyc02g067410 | VvHB07 | GSVIVT01019655001 | Oshox6 | Os09g35910 | Zmhdz3 | GRMZM2G003304 | Gmhdz4 | Glyma01g38390 |
| ATHB6 | AT2G22430 | SLZH10 | Solyc02g077590 | VvHB18 | GSVIVT01019012001 | Oshox8 | Os10g23090 | Zmhdz4 | GRMZM2G351330 | Gmhdz8 | Glyma02g02290 |
| ATHB7 | AT2G46680 | SLZH12 | Solyc02g086930 | VvHB27 | GSVIVT01003431001 | Oshox12 | Os03g10210 | Zmhdz5 | GRMZM2G178741 | Gmhdz9 | Glyma02g02630 |
| ATHB12 | AT3G61890 | SLZH13 | Solyc02g087840 | VvHB31 | GSVIVT01033744001 | Oshox13 | Os03g08960 | Zmhdz6 | GRMZM2G117164 | Gmhdz10 | Glyma02g06560 |
| ATHB13 | AT1G69780 | SLZH16 | Solyc03g034110 | VvHB50 | GSVIVT01032491001 | Oshox14 | Os07g39320 | Zmhdz7 | GRMZM2G002915 | Gmhdz15 | Glyma03g34710 |
| ATHB16 | AT4G40060 | SLZH17 | Solyc03g034120 | VvHB51 | GSVIVT01011377001 | Oshox16 | Os02g49700 | Zmhdz8 | GRMZM2G056600 | Gmhdz18 | Glyma04g34341 |
| ATHB20 | AT3G01220 | SLZH18 | Solyc03g034130 | VvHB54 | GSVIVT01027407001 | Oshox20 | Os08g37580 | Zmhdz9 | GRMZM2G041462 | Gmhdz19 | Glyma04g40960 |
| ATHB21 | AT2G18550 | SLZH19 | Solyc03g034150 | VvHB56 | GSVIVT01038619001 | Oshox21 | Os03g07450 | Zmhdz10 | GRMZM2G041127 | Gmhdz20 | Glyma05g01390 |
| ATHB22 | AT2G36610 | SLZH20 | Solyc03g082550 | VvHB58 | GSVIVT01008065001 | Oshox22 | Os04g45810 | Zmhdz11 | GRMZM2G139963 | Gmhdz24 | Glyma05g30940 |
| ATHB23 | AT1G26960 | SLZH21 | Solyc03g113270 | VvHB62 | GSVIVT01009083001 | Oshox23 | Os10g26500 | Zmhdz12 | GRMZM2G034113 | Gmhdz27 | Glyma06g13890 |
| ATHB40 | AT4G36740 | SLZH24 | Solyc04g005800 | VvHB70 | GSVIVT01014276001 | Oshox24 | Os02g43330 | Zmhdz13 | GRMZM2G097349 | Gmhdz28 | Glyma06g20230 |
| ATHB51 | AT5G03790 | SLZH25 | Solyc04g074700 |  |  | Oshox25 | Os09g21180 | Zmhdz14 | GRMZM2G132367 | Gmhdz32 | Glyma07g05800 |
| ATHB52 | AT5G53980 | SLZH27 | Solyc05g006980 |  |  |  |  | Zmhdz15 | GRMZM2G005624 | Gmhdz38 | Glyma08g14130 |
| ATHB53 | AT5G66700 | SLZH28 | Solyc05g007180 |  |  |  |  | Zmhdz16 | GRMZM2G119999 | Gmhdz43 | Glyma08g40705 |
| ATHB54 | AT1G27045 | SLZH30 | Solyc05g051460 |  |  |  |  | Zmhdz17 | AC233899.1_FGP004 | Gmhdz44 | Glyma08g40970 |
|  |  | SLZH32 | Solyc06g053220 |  |  |  |  |  |  | Gmhdz51 | Glyma09g37410 |
|  |  | SLZH36 | Solyc07g062790 |  |  |  |  |  |  | Gmhdz60 | Glyma11g37920 |
|  |  | SLZH41 | Solyc08g083130 |  |  |  |  |  |  | Gmhdz65 | Glyma13g05270 |
|  |  | SLZH42 | Solyc09g008810 |  |  |  |  |  |  | Gmhdz66 | Glyma13g23890 |
|  |  | SLZH48 | Solyc11g010270 |  |  |  |  |  |  | Gmhdz72 | Glyma16g02390 |
|  |  |  |  |  |  |  |  |  |  | Gmhdz75 | Glyma17g10490 |
|  |  |  |  |  |  |  |  |  |  | Gmhdz78 | Glyma18g01830 |
|  |  |  |  |  |  |  |  |  |  | Gmhdz79 | Glyma18g15970 |
|  |  |  |  |  |  |  |  |  |  | Gmhdz80 | Glyma18g16390 |
|  |  |  |  |  |  |  |  |  |  | Gmhdz83 | Glyma18g49290 |
|  |  |  |  |  |  |  |  |  |  | Gmhdz84 | Glyma19g01300 |
|  |  |  |  |  |  |  |  |  |  | Gmhdz85 | Glyma19g02490 |
|  |  |  |  |  |  |  |  |  |  | Gmhdz87 | Glyma19g37380 |
| **Total: 17** | | **Total: 22** | | **Total: 13** | | **Total: 14** | | **Total: 17** | | **Total: 30** | |
