# Supplementary figures and images for: The grapevine homeobox gene VvHB58 influences seed and fruit development through multiple hormonal signaling pathways
Source: BMC Plant Biol. 2019 Nov 27;19:523. doi: 10.1186/s12870-019-2144-9 (PMC6882351; doi:10.1186/s12870-019-2144-9)

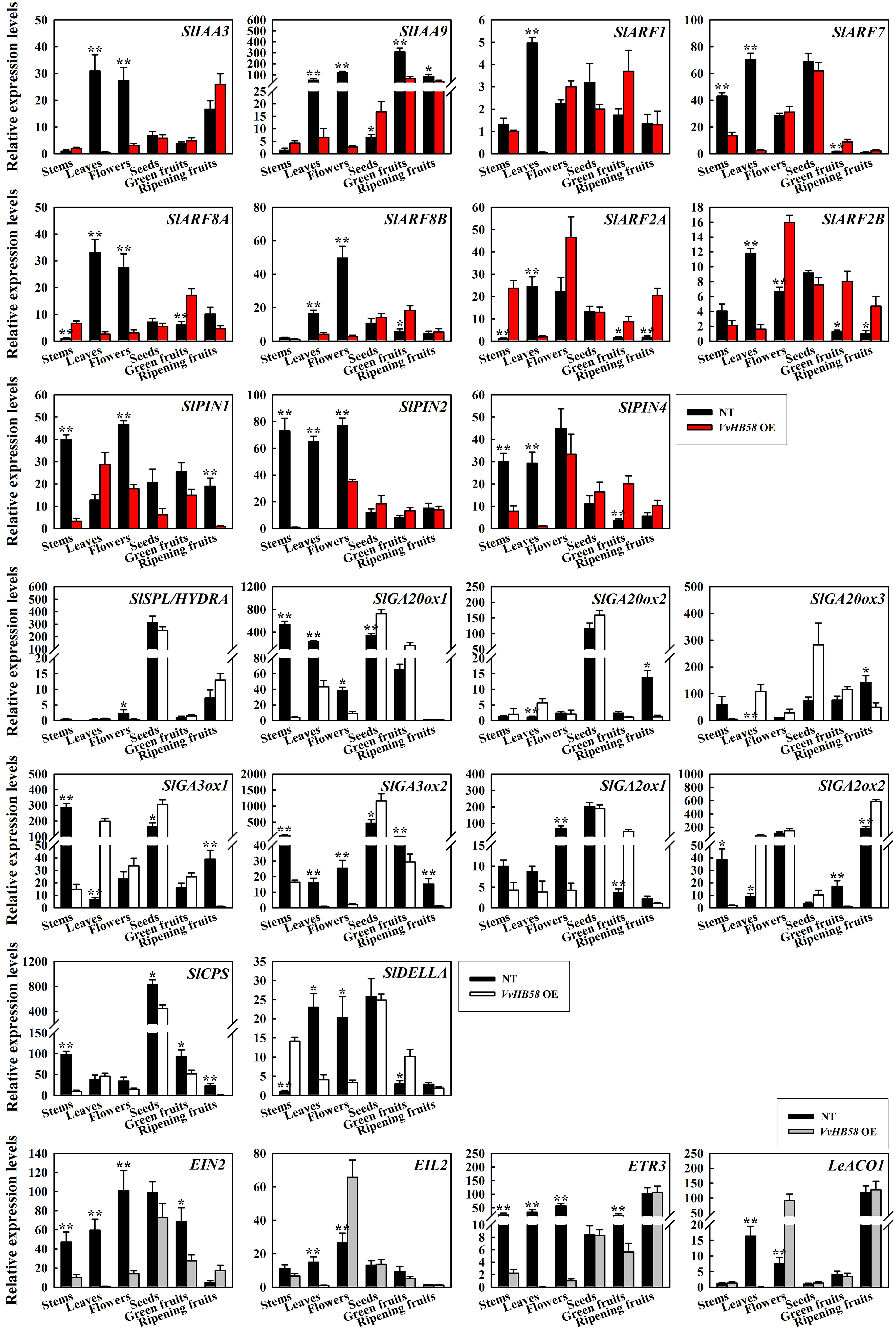

Supplement: Supplementary file 2 — Additional file 2: Figure S1. Transcript levels of auxin, gibberellin and ethylene responsive genes between VvHB58 transgenic and nontransgenic tomatoes using qPCR. Transcripts of SlACTIN gene were used as an endogenous control. NT represents nontransgenic tomatoes. Bars represent means ± SD from three biological replicates. Asterisks stand for statistical significance (*0.01 < P < 0.05, **P < 0.01, one-way ANOVA). [file 12870_2019_2144_MOESM2_ESM.jpg]

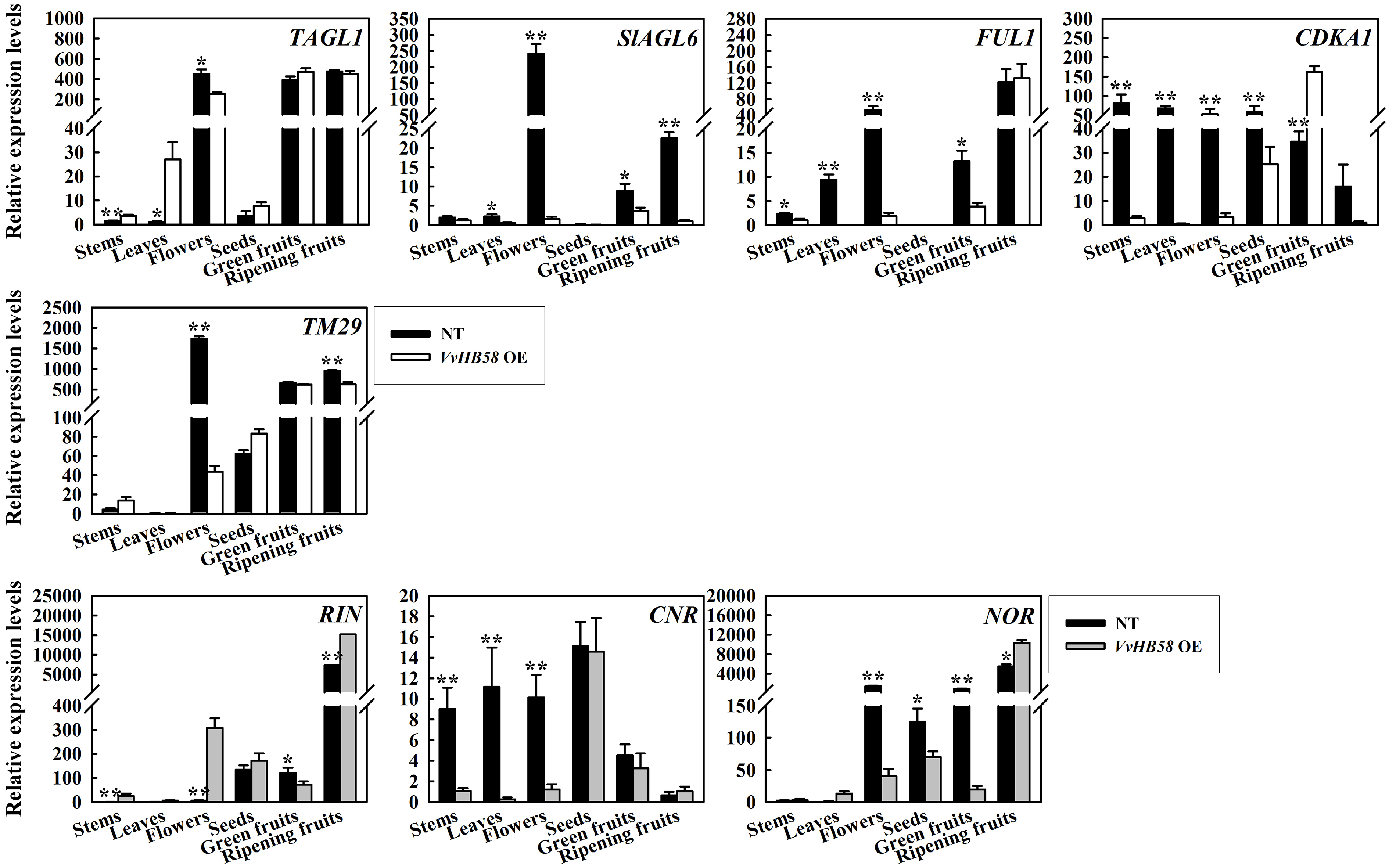

Supplement: Supplementary file 3 — Additional file 3: Figure S2. Expression analysis of seed development-related genes, and fruit ripening-related genes between VvHB58 transgenic and nontransgenic tomatoes via qPCR. Data were normalized to the expression levels of Solanum lycopersicum SlACTIN gene. NT represents nontransgenic tomatoes. Each value represents the means ± SD of three independent biological replicates. Significant differences were indicated by *0.01 < P < 0.05, **P < 0.01, one-way ANOVA. [file 12870_2019_2144_MOESM3_ESM.jpg]

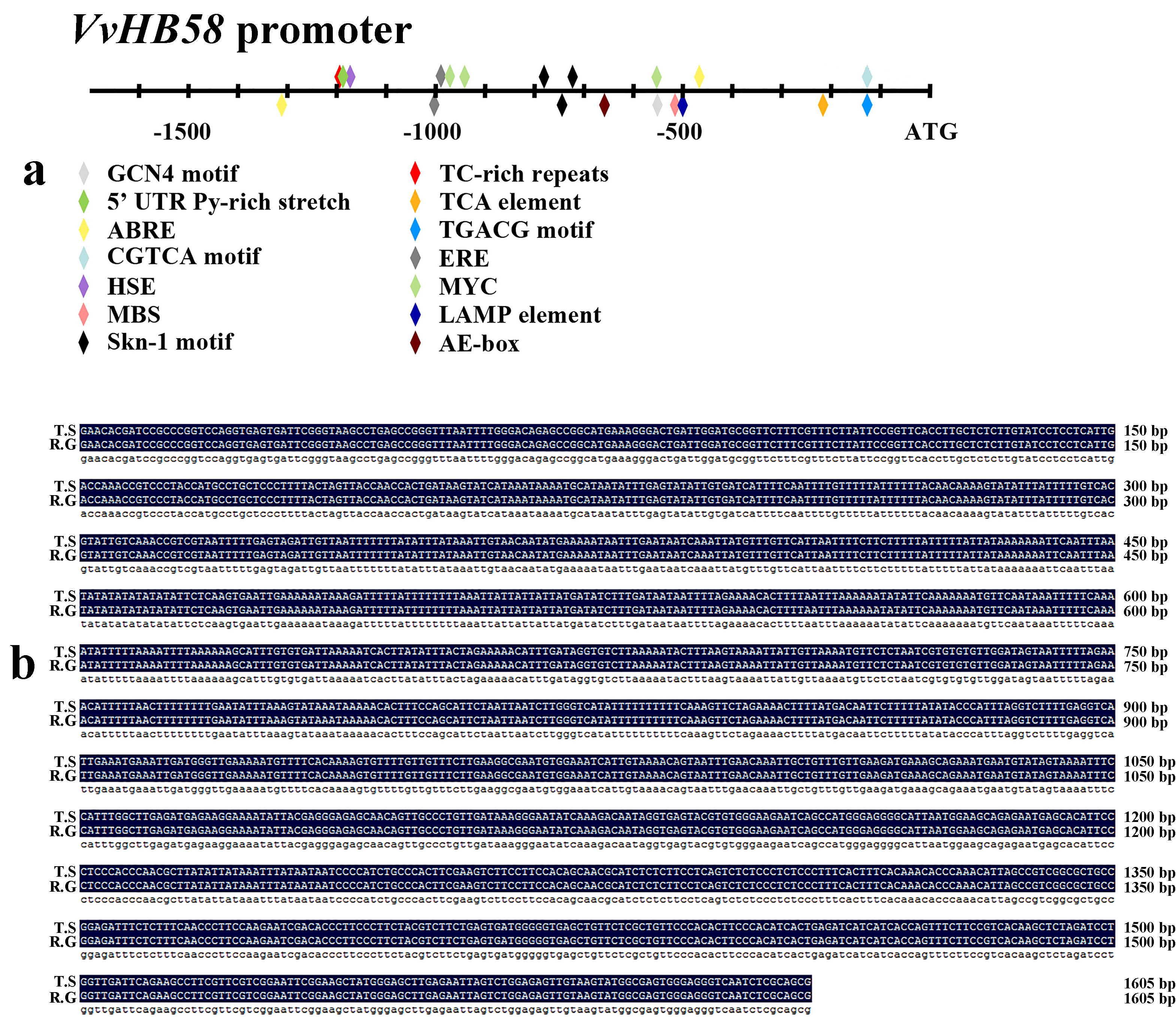

Supplement: Supplementary file 4 — Additional file 4: Figure S3. Analysis of cis-acting elements of VvHB58 promoter and alignment of promoter sequences obtained from seedless grape (Thompson Seedless, T.S) and seeded grape (Red Globe, R.G). [file 12870_2019_2144_MOESM4_ESM.jpg]

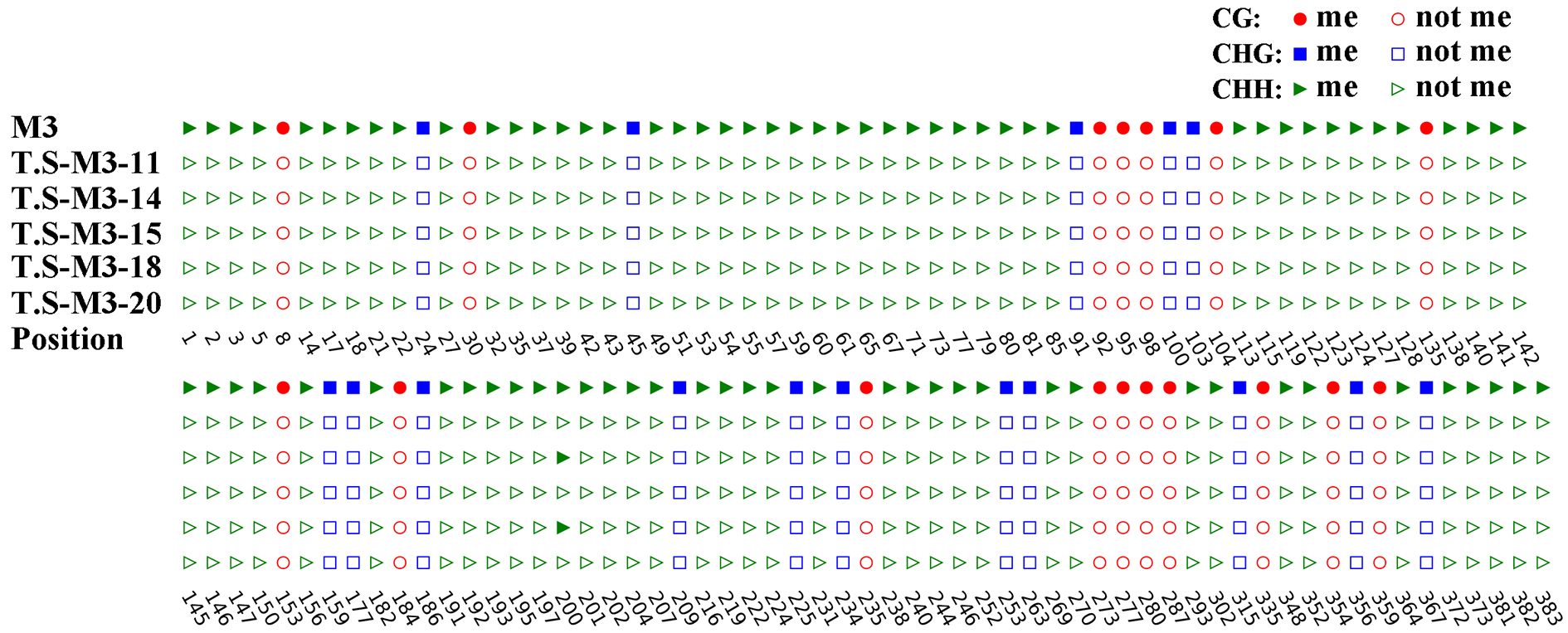

Supplement: Supplementary file 6 — Additional file 6: Figure S4. Analysis of DNA methylation in M3 region of VvHB58 gene in ‘Thompson Seedless’ grape using bisulfite sequencing. Red, blue, and green stand for three types of cytosine methylation symmetric CG, symmetric CHG, and asymmetric CHH (H = A, C, or T) respectively. Representative sequencing results of 5 single colonies were shown. [file 12870_2019_2144_MOESM6_ESM.jpg]

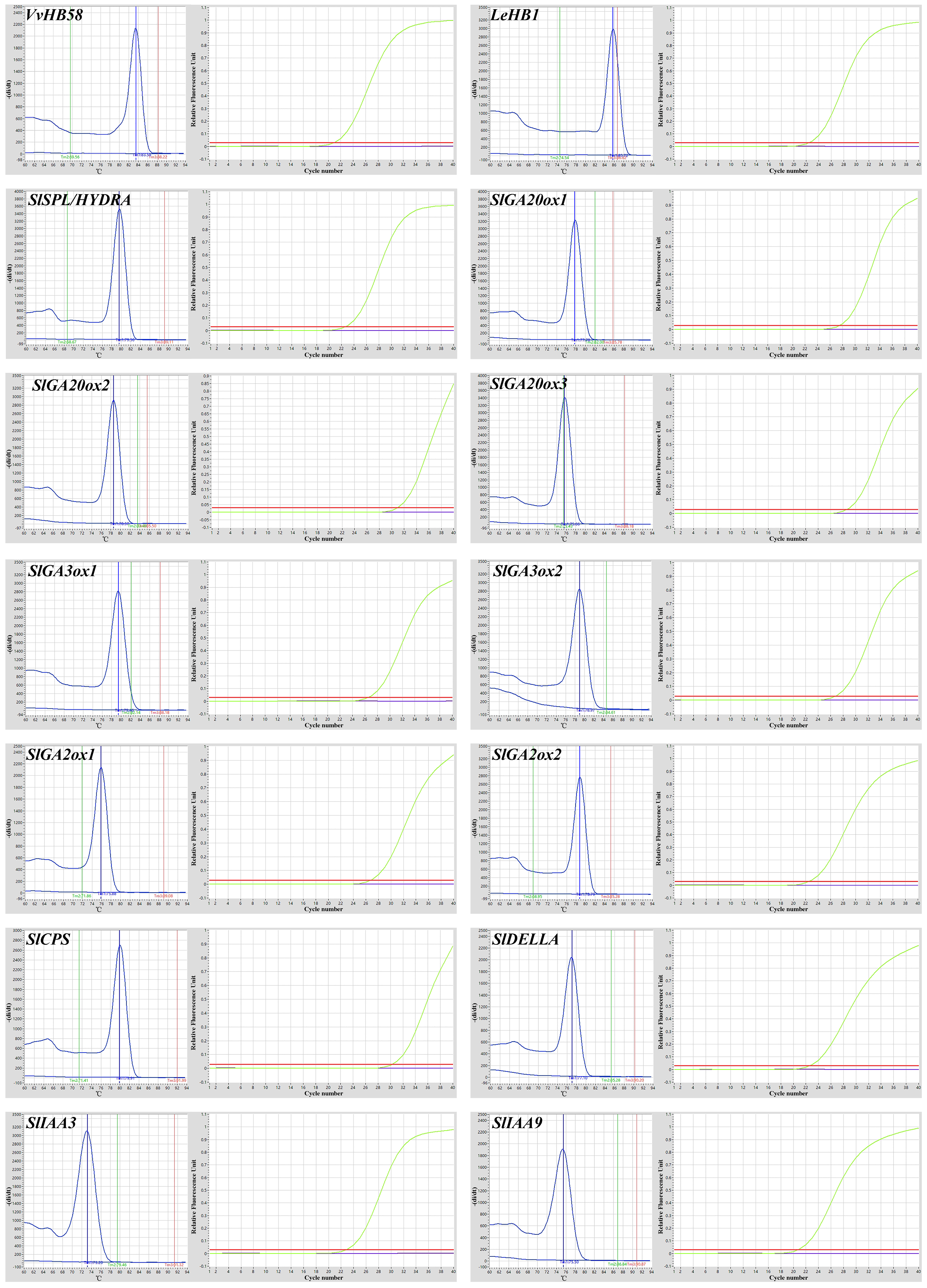

Supplement: Supplementary file 7 — Additional file 7: Figure S5. The melting curve of each gene used in qPCR. The melting curve of each gene was a single peak. In the amplification plot, the purple line represents the negative control and the green line represents the sample analysis. [file 12870_2019_2144_MOESM7_ESM.jpg]

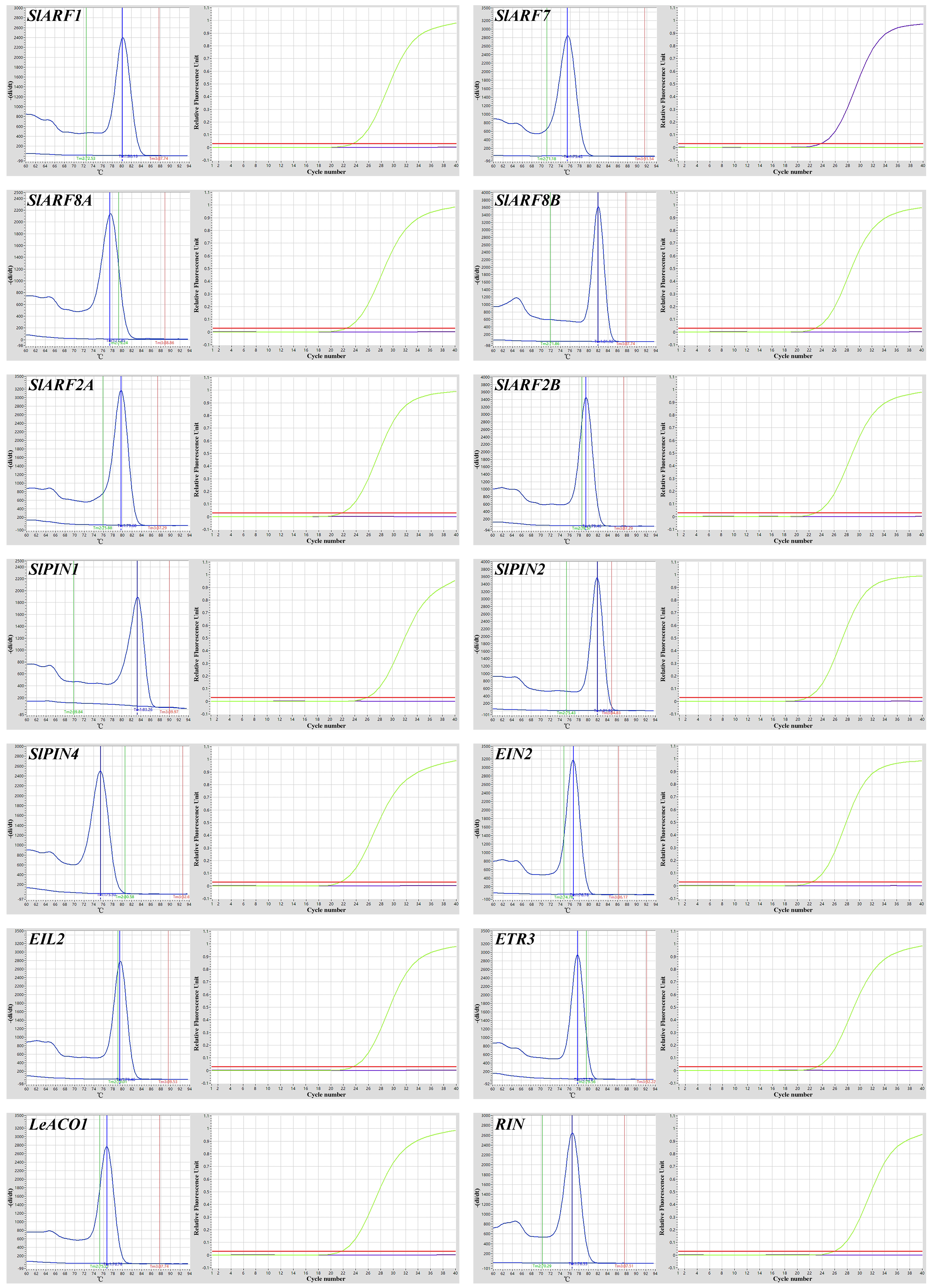

Supplement: Supplementary file 8 — Additional file 8: Figure S6. The melting curve of each gene used in qPCR. [file 12870_2019_2144_MOESM8_ESM.jpg]

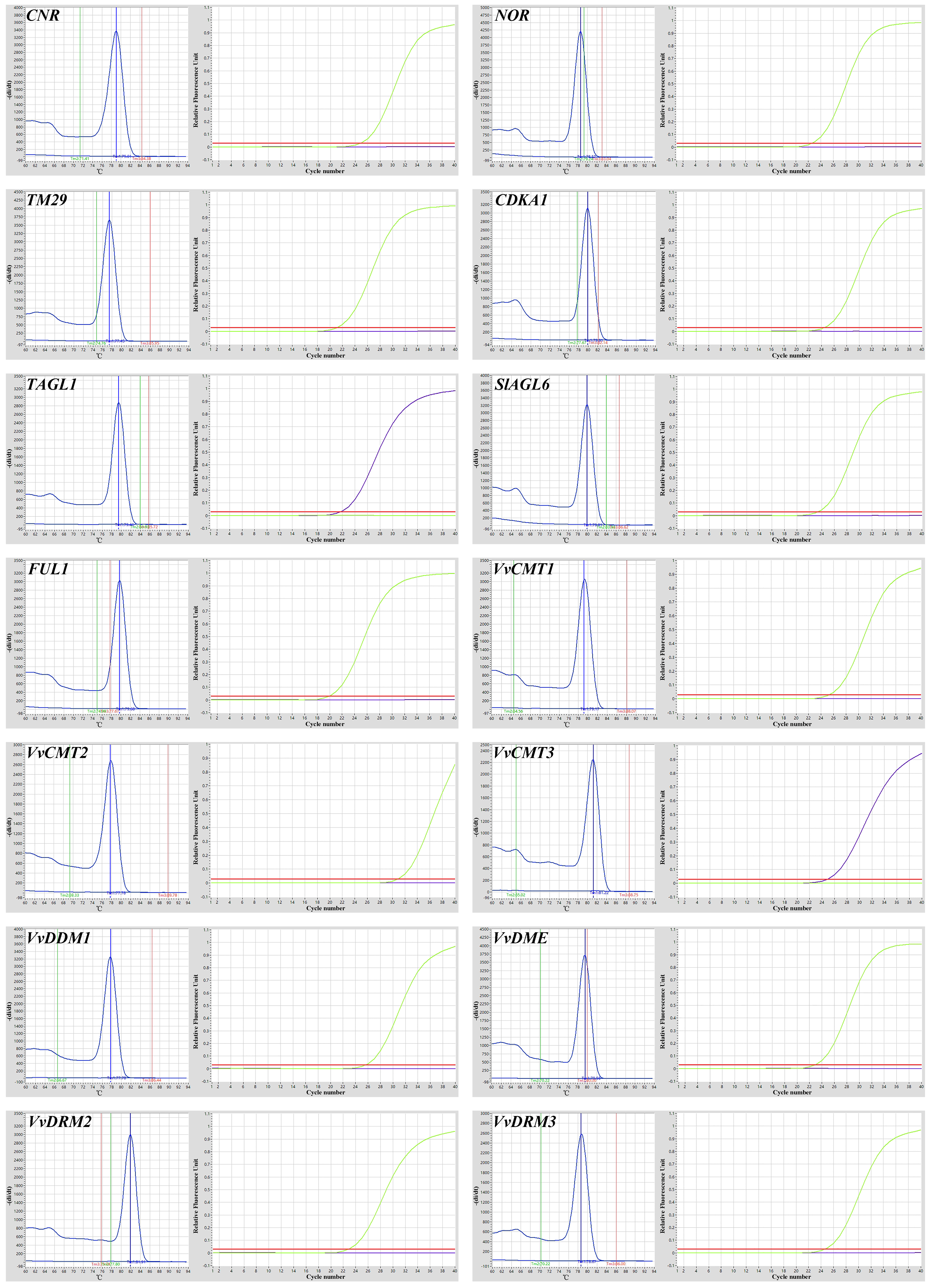

Supplement: Supplementary file 9 — Additional file 9: Figure S7. The melting curve of each gene used in qPCR. [file 12870_2019_2144_MOESM9_ESM.jpg]

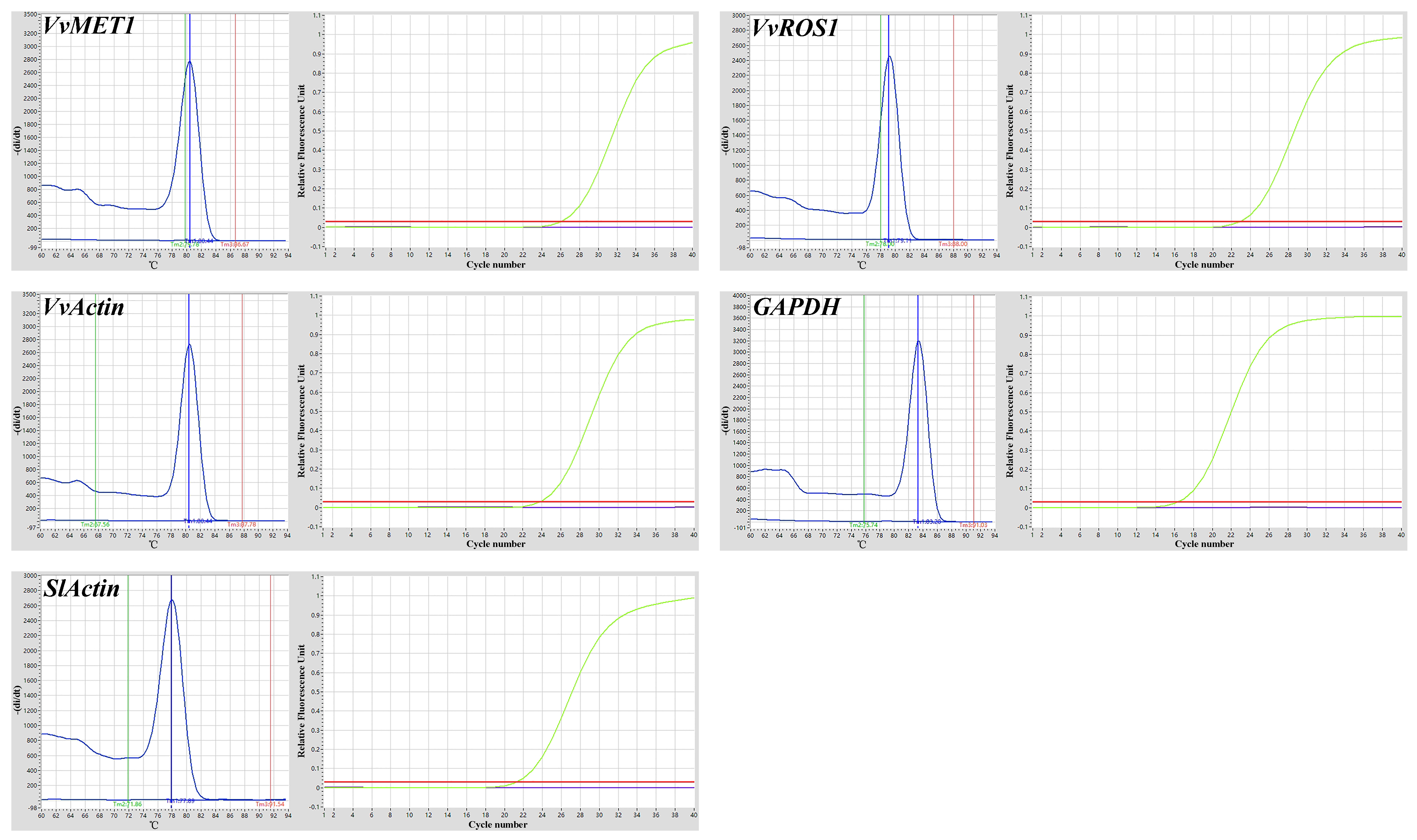

Supplement: Supplementary file 10 — Additional file 10: Figure S8. The melting curve of each gene used in qPCR. [file 12870_2019_2144_MOESM10_ESM.jpg]
