# Supplementary material for: The grapevine homeobox gene VvHB58 influences seed and fruit development through multiple hormonal signaling pathways
Source: BMC Plant Biol. 2019 Nov 27;19:523. doi: 10.1186/s12870-019-2144-9 (PMC6882351; doi:10.1186/s12870-019-2144-9)
Supplement: Supplementary file 5 — Additional file 5: Table S2. Homologs of DNA methyltransferase and demethylase proteins in grape. [file 12870_2019_2144_MOESM5_ESM.doc]

**Additional file 5: Table S2.** Homologs of DNA methyltransferase and demethylase proteins in grape.

| **Protein** | ***Arabidopsis thaliana* (GenBank)** | **Length** | **Gene name** | ***Vitis vinifera* (GenBank)** | **Length** |
| --- | --- | --- | --- | --- | --- |
| MET1 | AAA32829.1/AT5G49160 | 1534 aa | VvMET1 | [XP_002267200.1](https://www.ncbi.nlm.nih.gov/protein/XP_002267200.1?report=genbank&log$=prottop&blast_rank=1&RID=HEJYCDK101R) | 1549 aa |
| CMT1 | AEE36442.1/AT1G80740 | 791 aa | VvCMT1 | [XP_002275932.1](https://www.ncbi.nlm.nih.gov/protein/XP_002275932.1?report=genbank&log$=prottop&blast_rank=1&RID=HEN322AW014) | 829 aa |
| CMT2 | AAK69757.1/AT4G19020 | 1244 aa | VvCMT2 | [XP_019080798.1](https://www.ncbi.nlm.nih.gov/protein/XP_019080798.1?report=genbank&log$=prottop&blast_rank=1&RID=HENGWV8M014) | 1364 aa |
| CMT3 | AAK69756.1/AT1G69770 | 839 aa | VvCMT3 | [XP_010651344.1](https://www.ncbi.nlm.nih.gov/protein/XP_010651344.1?report=genbank&log$=prottop&blast_rank=1&RID=HENXER3U015) | 965 aa |
| DRM1 | AED92154.1/AT5G15380 | 624 aa | VvDRM2 | [XP_010660894.1](https://www.ncbi.nlm.nih.gov/protein/XP_010660894.1?report=genbank&log$=prottop&blast_rank=1&RID=HEP8WU2T014) | 602 aa |
| DRM2 | AED92056.1/AT5G14620 | 626 aa | VvDRM2 | [XP_010660894.1](https://www.ncbi.nlm.nih.gov/protein/XP_010660894.1?report=genbank&log$=prottop&blast_rank=1&RID=HEP8WU2T014) | 602 aa |
|  |  |  | VvDRM3 | [XP_019075700.1](https://www.ncbi.nlm.nih.gov/protein/XP_019075700.1?report=genbank&log$=prottop&blast_rank=3&RID=HEPT89CA014) | 589 aa |
| DDM1 | AAD28303.1/AT5G66750 | 764 aa | VvDDM1 | [XP_002267239.2](https://www.ncbi.nlm.nih.gov/protein/XP_002267239.2?report=genbank&log$=prottop&blast_rank=1&RID=HES8GM4H015) | 759 aa |
| DME | AED90760.1/AT5G04560 | 1729 aa | VvDME | [XP_002277401.1](https://www.ncbi.nlm.nih.gov/protein/XP_002277401.1?report=genbank&log$=prottop&blast_rank=2&RID=HF4DS13Z014) | 1942 aa |
| ROS1 | AAP37178.1/AT2G36490 | 1393 aa | VvROS1 | [CBI30244.3](https://www.ncbi.nlm.nih.gov/protein/CBI30244.3?report=genbank&log$=prottop&blast_rank=1&RID=HF4DS13Z014) | 1470 aa |
