# Supplementary material for: The grapevine homeobox gene VvHB58 influences seed and fruit development through multiple hormonal signaling pathways
Source: BMC Plant Biol. 2019 Nov 27;19:523. doi: 10.1186/s12870-019-2144-9 (PMC6882351; doi:10.1186/s12870-019-2144-9)
Supplement: Supplementary file 11 — Additional file 11: Table S3. Primers used for qPCR. In the qPCR reaction, the amplification temperature of these genes were 60 °C. SGN: Sol Genomics Network (https://solgenomics.net/). [file 12870_2019_2144_MOESM11_ESM.doc]

**Additional file 11: Table S3.** Primers used for qPCR. In the qPCR reaction, the amplification temperature of these genes were 60 ℃. SGN: Sol Genomics Network (https://solgenomics.net/).

| ***Gene*** | **Accession number** | **Amplicon length** | **Melting temperature** | **Forward primer (5'-3')** | **Reverse primer (5'-3')** |
| --- | --- | --- | --- | --- | --- |
| *VvHB58* | CBI15277 (GenBank) | 218 | 83.19 | GAGAGGGGAAACTTGGAGGTGT | TTTGTGGGAGGGTCGGGGTAAT |
| *LeHB1* | Solyc02g086930 (SGN) | 266 | 85.44 | CGAGGAATACTACGACGAGCA | CGGAGTCAAAATCAGAGAGAAGGG |
| *SlSPL/HYDRA* | KX023800 (GenBank) | 126 | 78.61 | ATCATCACCCTATTCCTCAG | GTTCAACTCCCATTCCTCTT |
| *SlGA20ox1* | [AF049898](http://www.plantphysiol.org/lookup/external-ref?link_type=GEN&access_num=AF049898&atom=/plantphysiol/153/2/851.atom) (GenBank) | 138 | 77.79 | CTCATTGTGATCCAACATCATTAACA | CCATAAATGTGTCGCCTATATTAACC |
| *SlGA20ox2* | [AF049899](http://www.plantphysiol.org/lookup/external-ref?link_type=GEN&access_num=AF049899&atom=/plantphysiol/153/2/851.atom) (GenBank) | 159 | 78.46 | AAGAAGGATAAGGTGGTGAGG | CTGTTGAAGCCAAGTAGAGAAG |
| *SlGA20ox3* | [AF049900](http://www.plantphysiol.org/lookup/external-ref?link_type=GEN&access_num=AF049900&atom=/plantphysiol/153/2/851.atom) (GenBank) | 144 | 75.54 | TTGTCCCAAGAAAGATAAGGTGATT | GTTTGTAGGGTGTTCATATCAGCTCTATAT |
| *SlGA3ox1* | AB010991 (GenBank) | 75 | 79.14 | CGCTCTCCTGATGGTGTCACT | TTCAGACCACATGAGCTTCGA |
| *SlGA3ox2* | AB010992 (GenBank) | 79 | 78.69 | CGGGTTGTACCCGAGTGTTC | CCCATATAGATAGGCCACTGAAAGA |
| *SlGA2ox1* | EF441351 (GenBank) | 149 | 75.54 | TGGAAGGTTTAAGAGTGTGAG | GATGATAAGGTTTGTGTGGTG |
| *SlGA2ox2* | EF441352 (GenBank) | 178 | 78.46 | ATTAAGATCCAATAACACTTCG | TCTTGATTTCACACTATTTGC |
| *SlCPS* | [AB015675](http://www.plantphysiol.org/lookup/external-ref?link_type=GEN&access_num=AB015675&atom=/plantphysiol/153/2/851.atom) (GenBank) | 151 | 79.59 | ATACCTAGAGCTAGCGAAATC | ACTGCCTAAATAGTACGTAACC |
| *SlDELLA* | Solyc11g011260 (SGN) | 81 | 76.66 | TCAACCTCCGGTGAACAAT | TCACAAGCCACCACGTTAC |
| *SlIAA3* | NM_001279100 (NCBI) | 51 | 72.61 | AAGTTAGCATGGATGGAGCACC | AAACCTGAAGATCAATTTTCCTCAA |
| *SlIAA9* | Solyc04g076850 (SGN) | 52 | 74.86 | GGCCACCCATTCGATCTTTT | TTCTTCGAGGCAGAGGCTAGA |
| *SlARF1* | Solyc01g103050 (SGN) | 83 | 79.59 | ACCCACGAGTCTCAAAGCAA | AGATCAACTGCCCTCCCAAC |
| *SlARF7* | Solyc07g042260 (SGN) | 51 | 74.86 | GCTGACACTGAAACTGATGAGGTC | GACAGGTTGAAGAGTCATCTGAGC |
| *SlARF8A* | Solyc03g031970 (SGN) | 51 | 76.66 | CCCGAGGATGTGCAGAAACT | GCGGTTTAGGGATCCAACCT |
| *SlARF8B* | Solyc02g037530 (SGN) | 195 | 81.17 | AACAGGCTCACCAACACACTTA | TATTACTTCCTTCAGAACACAACGA |
| *SlARF2A* | Solyc03g118290.2.1 (SGN) | 84 | 79.36 | GATGGTTCTAGGCCGGTTTC | CTCCGTGTACAACGCCGTAT |
| *SlARF2B* | Solyc12g042070.1.1 (SGN) | 99 | 79.36 | GCTTGTGACAGTGCCATGTG | TGCTGGTCTGAAGCTTGGTT |
| *SlPIN1* | AB508931 (GenBank) | 290 | 83.19 | TGTCTTTAGGGGAGGAGGTGA | GGCTGGAGTAAGTATTTGGGTTC |
| *SlPIN2* | AB508932 (GenBank) | 233 | 81.62 | GTGGAAAAGATGGCGATGAAGA | CCAAATCAGACCAATGAGGCTT |
| *SlPIN4* | XM_010322243.3 (NCBI) | 51 | 75.09 | TTTAGGCTGCATTGCCACAA | ACTCCTTAGCAAACACAAATGGG |
| *EIN2* | Solyc09g007870 (SGN) | 106 | 76.66 | GTGTGCTGAATAAGTTTAGTGGAG | TGCTGTACAATAGAAGAATGGAGG |
| *EIL2* | Solyc01g009170 (SGN) | 101 | 79.36 | TGAAGATGATGGAAGTCTGTAAGG | CCACTCCCTGAGATTATCCGA |
| *ETR3* | Solyc09g075440 (SGN) | 101 | 78.01 | TGCTGTTCGTGTACCGCTTT | TCATCGGGAGAACCAGAACC |
| *LeACO1* | Solyc07g049530 (SGN) | 82 | 76.66 | GCCAAAGAGCCAAGATTTGA | TTTTTAATTGAATTGGGATCTAAGC |
| *RIN* | Solyc05g012020 (SGN) | 64 | 76.44 | ATGCAGCACCATCAACACAT | CTCCAAATTCAAAGCATCCA |
| *CNR* | Solyc02g077850 (SGN) | 171 | 78.91 | GTGCCCACAAAACAACAAAGG | ATGGCGAGAGCAGACAAGC |
| *NOR* | Solyc10g006880 (SGN) | 198 | 78.69 | AGAGAACGATGCATGGAGGTTTGT | ACTGGCTCAGGAAATTGGCAATGG |
| *TM29* | AJ302015 (GenBank) | 134 | 77.56 | GTGGAAGGAGGGATGGGTAG | GCCTCACAAAGCACAGATAGTTC |
| *CDKA1* | [Y17225](https://www.ncbi.nlm.nih.gov/nucleotide/Y17225.1?report=genbank&log$=nuclalign&blast_rank=1&RID=0C5UY7B701R) (GenBank) | 224 | 79.36 | AACCCCTGAATAGAACCAAATG | GTATGTGCCGTGATTGTCTG |
| *TAGL1* | NP_001300859 (NCBI) | 186 | 79.14 | ACTTTCTGTTCTTTGTGATGCT | TTGGATGCTTCTTGCTGGTAG |
| *SlAGL6* | NP_001348459 (NCBI) | 144 | 79.59 | AAACCCTTGAGAGGTACCAACG | CACCAAGCAAGTGCCTTTGAG |
| *FUL1* | Solyc06g069430 (SGN) | 174 | 80.04 | TGACTATGCCAACGATTCCTG | ACATAATGCTTTTGGTTCCTCTG |
| *VvCMT1* | XP_002275932.1 (NCBI) | 132 | 79.14 | CAACCAGGCTATTATTCACC | GCATTGCCCACTTGTATGT |
| *VvCMT2* | XP_019080798.1 (NCBI) | 102 | 77.79 | CCAATACGACTTTCCCAGAGG | AGCCAGTTGACATTCCACCAC |
| *VvCMT3* | XP_010651344.1 (NCBI) | 257 | 80.72 | TTATGACCATCGCCCTCT | TTGGCACAGTCTCATCCC |
| *VvDDM1* | XP_002267239.2 (NCBI) | 170 | 77.34 | TATTGCGTCCCTTTCTCCTTCG | CCCAGTGCTTGCTTTCTCTT |
| *VvDME* | XP_002277401.1 (NCBI) | 374 | 79.49 | TGGATGGAAACCCTGATGTG | ATGGTGCTCTGTTCGTAGTCG |
| *VvDRM2* | XP_010660894.1 (NCBI) | 232 | 81.39 | TCTTCCGCTCCCTCCACTAAC | CCTTCCAACCCAGACCAAAT |
| *VvDRM3* | XP_019075700.1 (NCBI) | 141 | 78.91 | TCGTTTGCTTGAAATGGGTT | GTGGCTGAGGAGTGCTTGTCT |
| *VvMET1* | XP_002267200.1 (NCBI) | 133 | 80.49 | GAGGAGAGTTCAGACAAGGAG | CGTAATCAGCAACATCAGTGGAC |
| *VvROS1* | [CBI30244.3](https://www.ncbi.nlm.nih.gov/protein/CBI30244.3?report=genbank&log$=prottop&blast_rank=1&RID=HF4DS13Z014) (GenBank) | 180 | 79.36 | TGGTAGAGCAAGAGGTGGAAG | GAACGAGTAAGGTCATAGGCGAT |
| *VvActin* | AY680701 (GenBank) | 168 | 81.08 | GATTCTGGTGATGGTGTGAGT | GACAATTTCCCGTTCAGCAGT |
| *GAPDH* | CB973647 (GenBank) | 70 | 83.12 | TTCTCGTTGAGGGCTATTCCA | CCACAGACTTCATCGGTGACA |
| *SlActin* | NM_001321306 (NCBI) | 74 | 78.24 | TGTCCCTATTTACGAGGGTTATGC | CAGTTAAATCACGACCAGCAAGAT |
