# Supplementary material for: The grapevine homeobox gene VvHB58 influences seed and fruit development through multiple hormonal signaling pathways
Source: BMC Plant Biol. 2019 Nov 27;19:523. doi: 10.1186/s12870-019-2144-9 (PMC6882351; doi:10.1186/s12870-019-2144-9)
Supplement: Supplementary file 12 — Additional file 12: Table S4. Primers used for vector construction. [file 12870_2019_2144_MOESM12_ESM.doc]

**Additional file 12: Table S4.** Primers used for vector construction.

| **Primer name** | **Primers** | **Primer sequences (5'-3')** |
| --- | --- | --- |
| VvHB58: pCAMBIA2300 | F | GCTCTAGAATGCTTCAGAACCAAAGGGTCCCTT |
|  | R | GGGGTACCTCAATAGGACCAGGACCAAAAGGCA |
| VvHB58:pEearleyGate101 | F | GGGGACAAGTTTGTACAAAAAAGCAGGCTGCATGCTTCAGAACCAAAGG |
|  | R | GGGGACCACTTTGTACAAGAAAGCTGGGTCATAGGACCAGGACCAA |
