# Supplementary material for: The grapevine homeobox gene VvHB58 influences seed and fruit development through multiple hormonal signaling pathways
Source: BMC Plant Biol. 2019 Nov 27;19:523. doi: 10.1186/s12870-019-2144-9 (PMC6882351; doi:10.1186/s12870-019-2144-9)
Supplement: Supplementary file 13 — Additional file 13: Table S5. Primers used for detection of DNA methylation levels. [file 12870_2019_2144_MOESM13_ESM.doc]

Additional file 13: Table S5. Primers used for detection of DNA methylation levels.

| **Primer name** | **Product length** | **Primers** | **Primer sequences (5'-3')** |
| --- | --- | --- | --- |
| M1 | -1309bp ~ -898bp | F1 | GGTTTGAAAGGGATTGATTGG |
|  |  | F2 | TGAAAGGGATTGATTGGATG |
|  |  | R | CTAAAAATTACTATCCAACACACAC |
| M2 | -955bp ~ -499bp | F | TTATTAGAAAAATATTTGATAGGTG |
|  |  | R1 | TCTACTTCCATTAATACCCCTC |
|  |  | R2 | TTCTCTACTTCCATTAATACCCCTC |
| M3 | -360bp ~ 28bp | F1 | GGGTATTAATGGAAGTAGAGAATGAG |
|  |  | F2 | GTATTAATGGAAGTAGAGAATGAGT |
|  |  | R | AAACAAAAAACCCTTTAATTCTA |
